# AURKA emerges as a vulnerable target for KEAP1-deficient non-small cell lung cancer by activation of asparagine synthesis

**DOI:** 10.1038/s41419-024-06577-x

**Published:** 2024-03-23

**Authors:** Bing Deng, Fang Liu, Nana Chen, Xinhao Li, Jie Lei, Ning Chen, Jingjing Wu, Xuan Wang, Jie Lu, Mouxiang Fang, Ailin Chen, Zijian Zhang, Bin He, Min Yan, Yuchen Zhang, Zifeng Wang, Quentin Liu

**Affiliations:** 1 State Key Laboratory of Oncology in South China, Guangdong Provincial Clinical Research Center for Cancer, Sun Yat-sen University Cancer Center, Guangzhou, 510060 China; 2 Institute of Cancer Stem Cell, Dalian Medical University, Dalian, 116044 China; 3 Department of Oncology, The Seventh Affiliated Hospital, Sun Yat-sen University, Shenzhen, 518107 China; 4 MOE Key Laboratory of Laser Life Science & Institute of Laser Life Science, College of Biophotonics, South China Normal University, Guangzhou, 510631 China; 5 Department of Hematology, Guangdong Provincial People’s Hospital, Guangdong Academy of Medical Sciences, Guangzhou, 510080 China

**Keywords:** Targeted therapies, Non-small-cell lung cancer

## Abstract

AURKA is an established target for cancer therapy; however, the efficacy of its inhibitors in clinical trials is hindered by differential response rates across different tumor subtypes. In this study, we demonstrate AURKA regulates amino acid synthesis, rendering it a vulnerable target in KEAP1-deficient non-small cell lung cancer (NSCLC). Through CRISPR metabolic screens, we identified that KEAP1-knockdown cells showed the highest sensitivity to the AURKA inhibitor MLN8237. Subsequent investigations confirmed that KEAP1 deficiency heightens the susceptibility of NSCLC cells to AURKA inhibition both in vitro and in vivo, with the response depending on NRF2 activation. Mechanistically, AURKA interacts with the eIF2α kinase GCN2 and maintains its phosphorylation to regulate eIF2α-ATF4-mediated amino acid biosynthesis. AURKA inhibition restrains the expression of asparagine synthetase (ASNS), making KEAP1-deficient NSCLC cells vulnerable to AURKA inhibitors, in which ASNS is highly expressed. Our study unveils the pivotal role of AURKA in amino acid metabolism and identifies a specific metabolic indication for AURKA inhibitors. These findings also provide a novel clinical therapeutic target for KEAP1-mutant/deficient NSCLC, which is characterized by resistance to radiotherapy, chemotherapy, and targeted therapy.

## Introduction

Aurora kinase A (AURKA) is a crucial mitotic kinase, and its activation plays important roles in a variety of cancers [1, 2]. Our previous researches have demonstrated that elevated expression of AURKA in breast cancer promotes cell proliferation, invasion, stemness, and chemotherapy resistance [3–5]. Moreover, AURKA is also significantly upregulated and associated with poor prognosis in non-small cell lung cancer (NSCLC) [6]. Inhibition of AURKA leads to spindle formation abnormalities and mitotic defects, ultimately resulting in tumor cell death [7, 8]. Several AURKA inhibitors, including alisertib (MLN8237), danusertib, and ENMD-2076, have undergone clinical trials and shown certain antitumor effects in multiple tumor types [9–11]. However, the phase III clinical trial of MLN8237 in relapsed peripheral T-cell lymphoma was declared unsuccessful, as it did not show a survival advantage compared to the control chemotherapy drugs [12]. Consequently, the clinical application of AURKA inhibitors has faced stagnation.

Combining AURKA inhibitors with other drugs has emerged as a promising approach to enhance therapeutic outcomes [13–15]. In our previous research, we employed CRISPR/Cas9 screens based on the concept of “synthetic lethality” to identify combination targets for AURKA inhibitors. We have demonstrated that the concurrent administration of MLN8237 with the Haspin inhibitor CHR-6494, or OXPHOS inhibitors such as metformin synergistically inhibited the proliferation of breast cancer cells both in vitro and in vivo [16, 17]. Additionally, exploring new indications, which has been proved feasible for various anticancer drugs [18–20], becomes a new outlet for the clinical application of AURKA inhibitors. Therefore, identifying specific tumor subtypes or indications that exhibit sensitivity to AURKA inhibitors is crucial for the potential application of AURKA inhibitor monotherapy. Tumor cells undergo metabolic reprogramming, and AURKA has been implicated in the regulation of multiple metabolic pathways in tumors [6, 21, 22]. Nevertheless, the presence of specific metabolic indications for AURKA inhibitors remains unknown.

The E3 ligase KEAP1 is the primary negative regulator of the transcription factor NRF2 via mediating its protein degradation [23]. The KEAP1-NRF2 axis regulates multiple metabolic processes, including glutathione biosynthesis and circulation, NADPH regeneration and serine biosynthesis [24, 25]. Known as a tumor suppressor, KEAP1 mutation or deficiency leads to the activation of NRF2 and its downstream targets [26]. KEAP1 mutations have been observed in various cancers [27–30], with NSCLC exhibiting the highest mutation frequency up to 15% [26]. The KEAP1 mutations are not concentrated in specific hotspots but are rather distributed throughout the protein, encompassing missense, truncating, and other types of mutations [31, 32]. Besides, KEAP1 promoter methylation has been linked to poor prognosis in multiple cancers, including NSCLC [33], breast cancer [34], and malignant gliomas [35]. KEAP1 mutant NSCLC is highly refractory and resistant to traditional cancer treatments, such as radiotherapy [36], chemotherapy [37], and targeted therapy [38]. This resistance stems from their unique ability to counteract reactive oxygen species (ROS) due to enhanced antioxidant capacity [39]. Consequently, developing an effective clinical treatment strategy for this subtype requires further research. While the role of KEAP1 in tumor metabolism regulation is well-established [23, 24], there remains a need for further investigation into treating KEAP1-mutant or -deficient NSCLC by targeting specific metabolic features.

In this study, we employed CRISPR metabolic screens to find specific tumor subtypes harboring metabolism-related gene mutations that exhibit sensitivity to AURKA inhibitor MLN8237. Our findings reveal that MLN8237 is particularly effective in inhibiting the growth of KEAP1-mutant or -deficient tumor cells. Furthermore, we elucidated that inhibition of AURKA downregulates asparagine synthesis, thereby enhancing the sensitivity of KEAP1-deficient NSCLC to MLN8237. Consequently, our study unveils a promising druggable target for KEAP1-mutant or -deficient tumors and explores a novel clinical application for AURKA inhibitors.

## Results

### CRISPR/Cas9 metabolic screens identified KEAP1 as a significantly negatively selected gene for AURKA inhibition

**Fig. 1:**
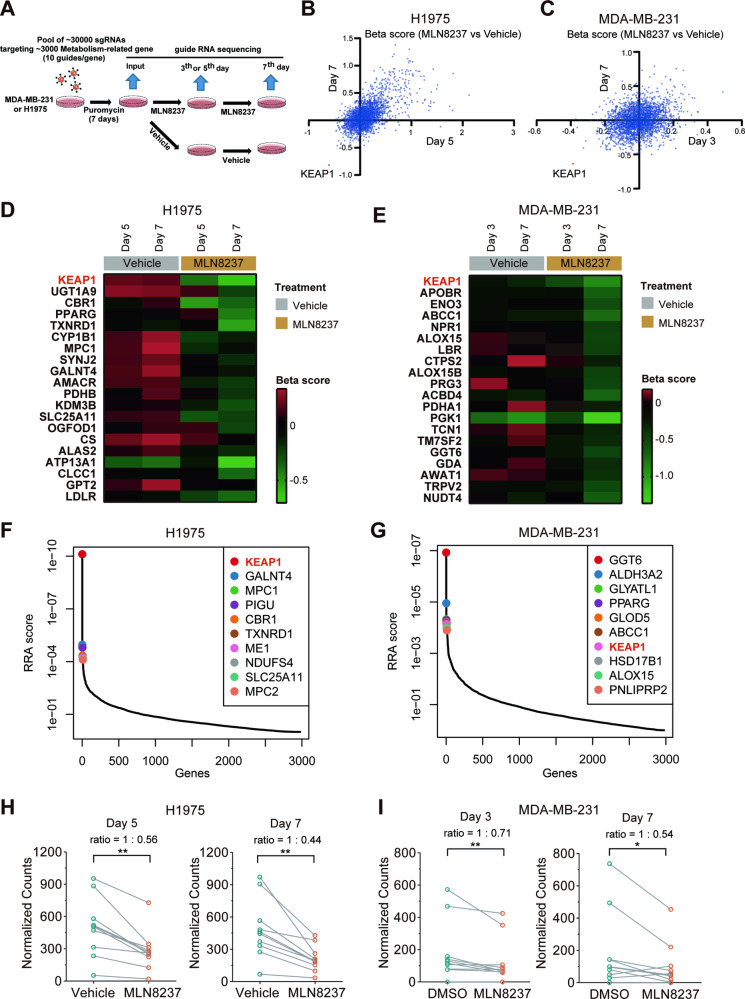
CRISPR/Cas9 metabolic screens identified KEAP1 as a significantly negatively selected gene for AURKA inhibition. **A** Schematic illustration of CRISPR/Cas9 metabolic screen in H1975 or MDA-MB-231 cells treated with MLN8237 or vehicle (DMSO). **B**, **C** The scatter plot of β scores reflecting the sgRNA enrichment in MLN8237-treated H1975 (**B**) or MDA-MB-231 (**C**) cells compared with the vehicle-treated cells at day 5 and day 7 **(B**) or day 3 and day 7 (**C**). KEAP1 is the highlighted with red as the most significant negatively selected gene in both plots. **D**, **E** Heatmap of β scores reflecting the sgRNA enrichment of the top 20 of negatively selected genes in H1975 cells (**D**) or MDA-MB-231 cells (**E**) with MLN8237 or vehicle treatment. **F**, **G** Ranking of the negatively selected genes in MLN8237-treated H1975 (F) or MDA-MB-231 (**G**) cells compared with the vehicle-treated cells at day 7 with robust rank aggregation (RRA) scores. **H**, **I** The normalized read counts of KEAP1 sgRNAs in H1975 cells (**H**) or MDA-MB-231 cells (**I**) treated with vehicle or MLN8237 at day 5 and day 7 (**H**) or day 3 and day 7 (**I**). The ratios of average normalized read counts in two treatment groups were calculated. Wilcoxon matched-pairs signed rank test was performed. *P < 0.05; **P < 0.01.

To identify the metabolic indications of AURKA inhibitors, we performed CRISPR/Cas9 screens using the metabolic library in lung and breast cancer cells (Fig. 1A). The metabolic library contains approximately 30000 sgRNAs targeting 2981 metabolism-related genes, with 10 sgRNAs assigned to each gene [40]. The model-based analysis of genome-wide CRISPR/Cas9 knockout (MAGeCK) [41] was applied to analyze the sequencing results (Supplementary Table 1). The histogram of median normalized read counts, the distribution of read counts in each group and the distinct evolutionary routes over time in vehicle (DMSO) or MLN8237-treated groups suggested that we obtained high-quality screen data in both cell lines (Fig. S1A, B).

To identify the specific metabolism-related gene whose knockout enhances sensitivity to MLN8237, we focused on the negatively selected genes in the MLN8237-treated groups compared with the vehicle-treated groups. Beta score analysis of the whole gene set or the top 20 negatively selected genes showed that KEAP1 was the most significantly negatively selected gene in both cell lines (Fig. 1B-E). The robust rank aggregation (RRA) scores and *p* values of negatively selected genes in the comparison between the MLN8237-treated groups and the vehicle-treated groups showed that KEAP1 was significantly negatively selected in both cell lines, especially in H1975 cells (Fig. 1F, G and Fig. S1C, D). Consistently, the normalized read counts of KEAP1 sgRNAs considerably decreased in the MLN8237-treated groups compared with the vehicle-treated groups in H1975 cells at both day 5 and day 7 (Fig. 1H), and in MDA-MB-231 cells at both day 3 and day 7 (Fig. 1I). All these results show that CRISPR/Cas9 metabolic screens have identified KEAP1 is a significantly negatively selected gene under the treatment of AURKA inhibitor MLN8237 in both MDA-MB-231 and H1975 cells.

### KEAP1 deficiency sensitizes NSCLC cells to AURKA inhibition through NRF2 activation

**Fig. 2:**
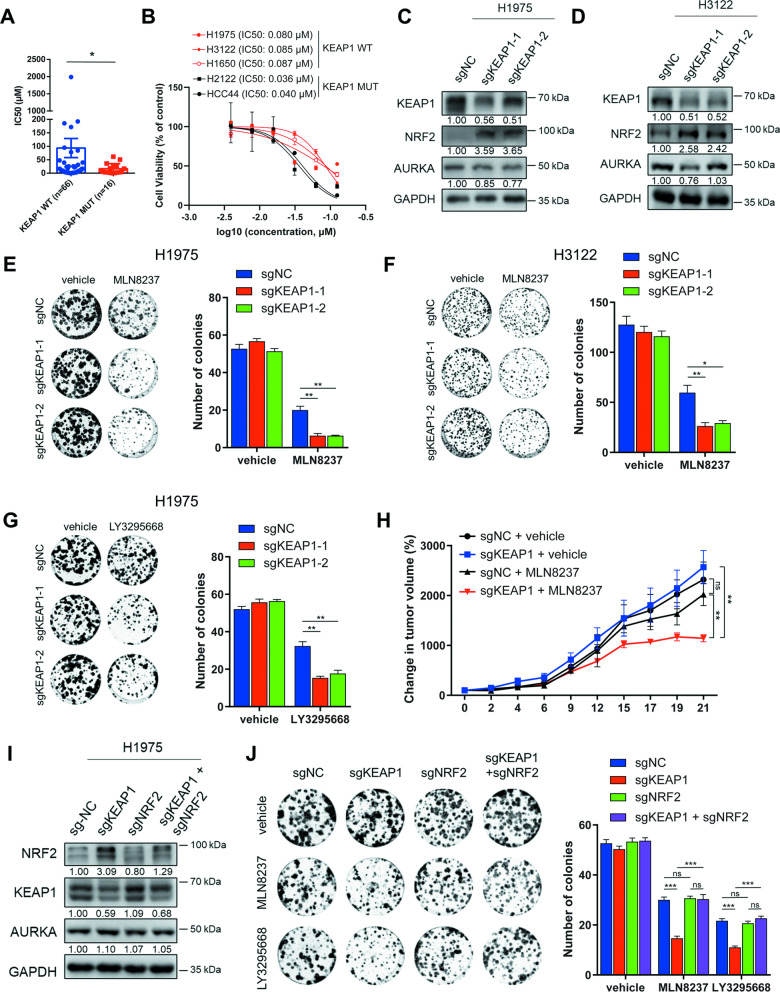
KEAP1 deficiency sensitizes NSCLC cells to AURKA inhibition through NRF2 activation. **A** The scatter plot with columns showing the IC50 of MLN8237 in KEAP1 wildtype (*n* = 66) and KEAP1 mutant (*n* = 16) NSCLC cell lines. **B** The cell viability of the KEAP1 wildtype NSCLC cell lines (H1975, H3122, H1650) and the KEAP1 mutant NSCLC cell lines (H2122, HCC44) (1000 cells/well) treated with the indicated doses of MLN8237 for 72 h. All groups were normalized to the vehicle group. **C**, **D** Western blot analysis (left) showing the knockdown effect of KEAP1 in H1975 cells (**C**) or H3122 cells **(D**). **E**, **F** Colony formation assays showing the cell viability of H1975-sgNC and H1975-sgKEAP1 cells (500 cells/well) treated with vehicle or 37.5 nM MLN8237 **(E**) or H3122-sgNC and H3122-sgKEAP1 cells (1000 cells/well) treated with vehicle or 50 nM MLN8237 (**F**). **G** Colony formation assays showing the cell viability of H1975-sgNC and H1975-sgKEAP1 cells (500 cells/well) treated with vehicle or 25 nM LY3295668. **H** The change in tumor volume after treatment with MLN8237 (30 mg/kg) or vehicle for the indicated time. Student t-tests were conducted on day 21 between the MLN8237 and vehicle treatment groups of H1975-sgNC or H1975-sgKEAP1 cells. **I** Western blot analysis showing the knockdown effect of NRF2 in H1975 cells with or without KEAP1 knockdown. **J** Colony formation assays showing the cell viability of H1975-sgNC, H1975-sgNRF2 and H1975-sgKEAP1 cells (500 cells/well) with or without NRF2 knockdown, in the condition of treatment with vehicle or 37.5 nM MLN8237 or 37.5 nM LY3295668. In **E**-**G** and **J**, the representative pictures of colonies are showed in left, and the number of colonies are counted in right. In **B-G**, **I** and **J**, the experiments were performed in three independent replicates. Statistics, significance: one-way ANOVA (alpha=0.05) with Bonferroni correction (**E**, **F**, **G**, **J**); significance: two-tailed unpaired t-test (**A**, **H**); Error bars, SEM; ns not significant; *P < 0.05; **P < 0.01; ***P < 0.001.

Given the high mutation frequency of KEAP1 in NSCLC, our focus was primarily on this cancer type to identify specific indications for AURKA inhibitors. To validate the sensitizing effect of KEAP1 mutation or deficiency on AURKA inhibitors, we initially analyzed the sensitivity of NSCLC cell lines with wildtype or mutant KEAP1 to AURKA inhibitors in the Cancer Drug Sensitivity Genomics database GDSC. Based on this information, we categorized the 82 NSCLC cell lines into two groups: KEAP1 wildtype and mutant (Supplementary Table 2). Analysis of cell line drug sensitivity in the GDSC database revealed that KEAP1 mutant cell lines exhibited significantly higher sensitivity to MLN8237 compared to KEAP1 wildtype cell lines (Fig. 2A).

Subsequently, we selected the KEAP1 wildtype NSCLC cell lines H1975, H3122, H1650, and the KEAP1 mutant NSCLC cell lines H2122 (F211C), HCC44 (A170-R204del) for further validation using CCK8 assays. The cell viability at different concentrations of MLN8237 in these cell lines also confirmed KEAP1 mutation sensitized the NSCLC cells to MLN8237 (Fig. 2B). Additionally, we conducted colony formation assays after KEAP1 knockdown in KEAP1 wildtype NSCLC cell lines H1975 and H3122 and observed a significant enhancement in sensitivity to AURKA inhibitors in both cell lines (Fig. 2C-G and Fig. S2A-C). The similar results were also obtained in MDA-MB-231 cells (Fig. S2D-G). Finally, the subcutaneous tumor formation experiments in nude mice using the H1975 wildtype and KEAP1-knockdown cells further confirmed that the sensitivity of NSCLC cells to the AURKA inhibitor MLN8237 was significantly enhanced after KEAP1 knockdown (Fig. 2H).

To investigate whether NRF2 activation mediate the sensitization effect of KEAP1 knockdown to AURKA inhibitors, we knocked down NRF2 in KEAP1-deficient cells (Fig. 2I). The colony formation results confirmed the mitigating effect of NRF2 knockdown on the sensitization of KEAP1 knockdown to AURKA inhibitors (Fig. 2J). Additionally, NRF2 knockdown in the KEAP1-mutant cell line HCC44 (KEAP1-F211C) significantly reduced the sensitivity of the cells to AURKA inhibitors (Fig. S2H-J). Collectively, KEAP1 deficiency enhances the sensitivity of the NSCLC cells to AURKA inhibition, and this effect depends on the activation of the NRF2 pathway.

### AURKA inhibition downregulates biosynthesis of amino acids

**Fig. 3:**
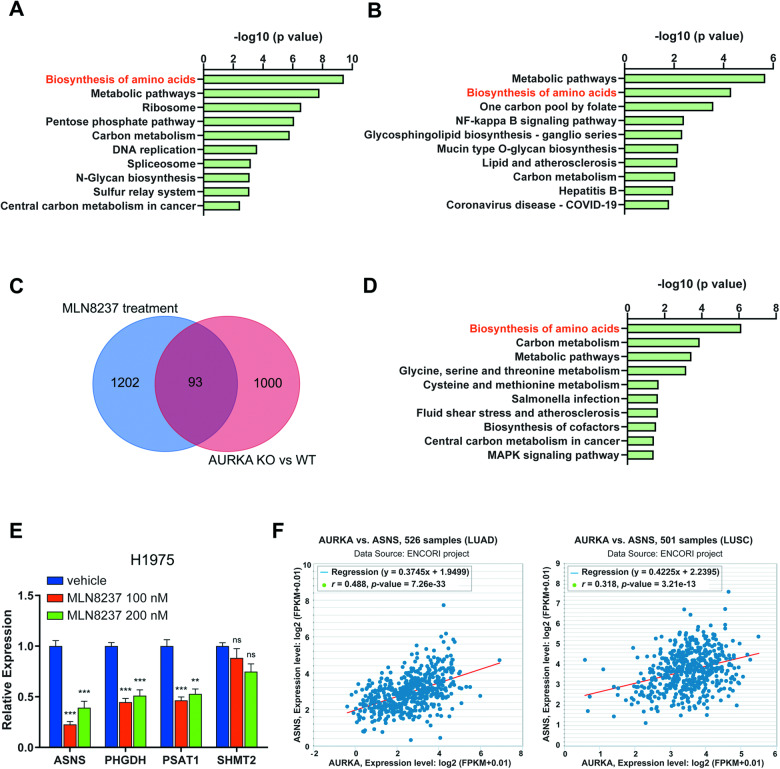
AURKA inhibition downregulates biosynthesis of amino acids. **A, B** KEGG pathway enrichment analysis of the downregulated genes in MLN8237-treated (**A**) or AURKA-knockout cells (**B**). **C** Venn plot showing the number of the overlapped downregulated genes in the two indicated groups. MLN8237 treatment: with 150 nM MLN8237 treatment for 72 h; AURKA KO vs WT: AURKA-knockout vs wildtype cells. **D** KEGG pathway enrichment analysis of 93 overlapped downregulated genes of the two groups in **C**. **E** The relative expression of the indicated genes of amino acid biosynthesis in H1975 cells (5 × 10^4^ cells/well) with treatment of the indicated doses of MLN8237 for 72 h in 12-well plate, determined by qPCR analysis. **F** Analysis of the co-expression patterns between AURKA and ASNS using the TCGA RNA-seq data of LUAD, LUSC from the starBase database. In **E**, the experiments were performed in three independent replicates. Statistics, significance: one-way ANOVA (alpha=0.05) with Bonferroni correction (**E**); Error bars, SEM; ns not significant; **P < 0.01; ***P < 0.001.

AURKA inhibition has been reported to induce changes in various pathways within tumors, and it can even trigger metabolic reprogramming in glioblastoma [22]. To investigate the concurrent alterations in signaling pathways resulting from AURKA inhibition and knockout, we conducted a KEGG pathway analysis on the downregulated genes identified from the RNA-seq data (Supplementary Table 3). The analysis revealed that the downregulated genes in both the MLN8237 treatment group and the AURKA knockout group, compared to the wildtype group, were significantly enriched in the pathway of biosynthesis of amino acids (Fig. 3A, B). Furthermore, the biosynthesis of amino acids pathway was also found to be the most significant pathway in the KEGG enrichment analysis using the genes common to both groups (Fig. 3C, D and Supplementary Table 4). These genes included ASNS, PHGDH, PSAT1, and SHMT2, suggesting that inhibition or knockout of AURKA leads to a notable decrease in amino acid synthesis.

Moreover, qPCR analysis confirmed that AURKA inhibition downregulates the expression of amino acid synthesis genes ASNS, PHGDH, and PSAT1 (Fig. 3E and Fig. S3A). Consistent with the RNA-seq data analysis, asparagine synthetase (ASNS) exhibited the most significant downregulation among the genes involved in the amino acid biosynthesis pathway under MLN8237 treatment or AURKA knockout (Fig. 3E and Fig. S3A, B). To further support these findings, we analyzed the TCGA RNA-seq data of NSCLC, including both lung adenocarcinoma (LUAD) and lung squamous cell carcinoma (LUSC), obtained from the starBase database [42]. This analysis confirmed a strong positive correlation between AURKA and ASNS expression in clinical samples (Fig. 3F). Consequently, our results suggest that AURKA plays a role in regulating the expression of asparagine synthetase (ASNS).

### KEAP1 deficiency intensifies AURKA inhibition-mediated eIF2α-ATF4-ASNS pathway downregulation and apoptosis

**Fig. 4:**
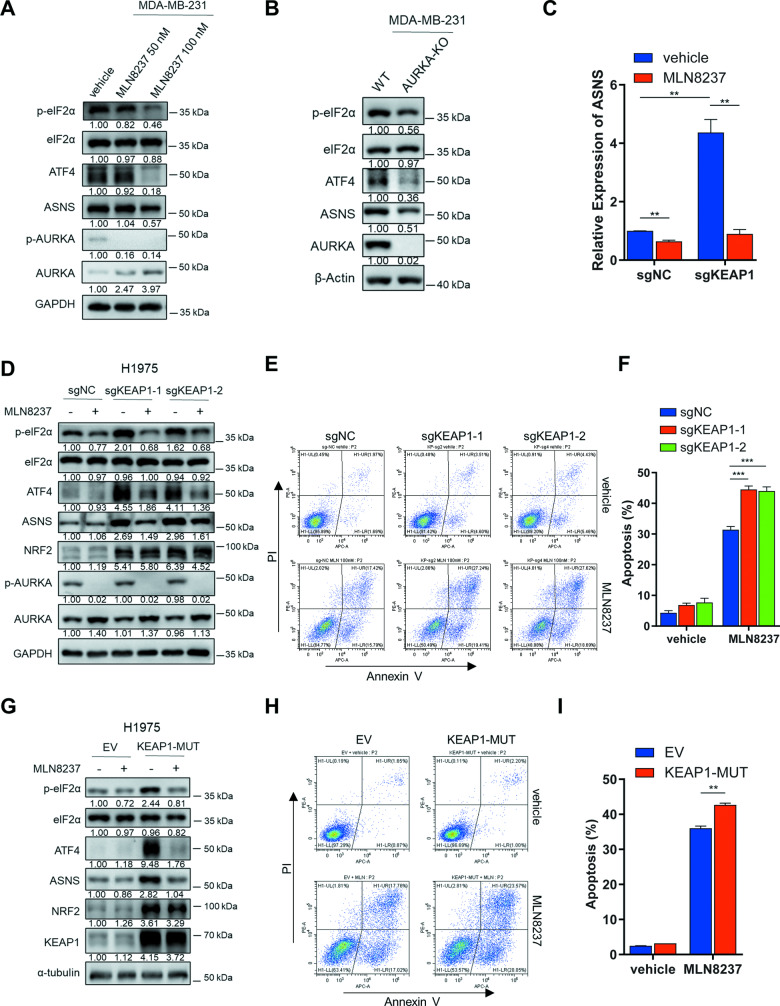
KEAP1 deficiency intensifies AURKA inhibition-mediated eIF2α-ATF4-ASNS pathway downregulation and apoptosis. **A, B** Western blot analysis revealing changes in the expression of the indicated proteins upon AURKA inhibition (**A**) or knockout (**B**). **C** The relative expression of ASNS determined by qPCR analysis in H1975-sgNC or sgKEAP1 cells (5 × 10^4^ cells/well) treated with 100 nM MLN8237 or vehicle for 72 h in 12-well plate. **D** Western blot analysis in H1975-sgNC or sgKEAP1 cells (5 × 10^4^ cells/well) treated with 50 nM MLN8237 or vehicle for 72 h in 12-well plate. **E** The represented graphs showing the percentage of apoptosis cells in H1975-sgNC and sgKEAP1 cells (5 × 10^4^ cells/well) treated with 100 nM MLN8237 or vehicle for 72 h. **F** The histogram showing the percentage of apoptosis cells in **E**. **G** Western blot analysis in H1975 cells overexpressing empty vector (EV) or mutant KEAP1 (KEAP1-MUT, F211C) (5 × 10^4^ cells/well) treated with 100 nM MLN8237 or vehicle for 72 h. **H** The represented graphs showing the percentage of apoptosis cells in **G**. **I** The histogram showing the percentage of apoptosis cells in H. All the experiments were performed in three independent replicates. Statistics, significance: one-way ANOVA (alpha=0.05) with Bonferroni correction (**F**); significance: two-tailed unpaired t-test (**C**, **I**); Error bars, SEM; ns not significant; **P < 0.01; ***P < 0.001.

The eIF2α-ATF4 axis has been reported to regulate the amino acid biosynthesis pathway [43, 44]. Additionally, it has been noted that oncogenic KRAS can disrupt amino acid homeostasis and asparagine biosynthesis through ATF4 regulation during nutrient depletion [45]. In order to elucidate the mechanism by which AURKA regulates ASNS expression, we investigated the impact of AURKA inhibition or knockout on the upstream regulatory pathway of ASNS. Western blot analysis was conducted on cells treated with MLN8237 or subjected to AURKA knockout, revealing a significant reduction in the levels of ASNS and its upstream regulatory components, p-eIF2α and ATF4 (Fig. 4A, B).

Subsequently, we examined the combined effects of KEAP1 deficiency and AURKA inhibition on the eIF2α-ATF4-ASNS pathway. Due to the heightened reliance on the asparagine synthesis pathway following KEAP1 knockdown, the expression of ASNS and its upstream regulators, p-eIF2α and ATF4, was markedly upregulated in KEAP1-knockdown cells (Fig. 4C, D). Importantly, treatment with AURKA inhibitor MLN8237, resulted in a more pronounced downregulation of ASNS, p-eIF2α, and ATF4 protein levels in KEAP1-knockdown cells, compared to KEAP1 wildtype cells (Fig. 4C, D). This suggests that AURKA inhibitors may exhibit enhanced growth inhibitory effects in KEAP1-deficient tumors by suppressing asparagine synthesis. Furthermore, KEAP1 knockdown increased the proportion of apoptotic cells in NSCLC cells treated with MLN8237 (Fig. 4E, F). To investigate the impact of KEAP1 mutation on AURKA inhibition, we generated stable H1975 cells overexpressing a mutated form of KEAP1 (KEAP1-F211C). We observed a notable activation of NRF2 in these cells, in line with previous reports underscoring the dominant-negative effect of mutant KEAP1 [28, 46]. As expected, the eIF2α-ATF4-ASNS pathway exhibited a significant downregulation, and there was an increase in the proportion of apoptotic cells when mutant KEAP1-overexpressing cells were treated with MLN8237 (Fig. 4G-I). Taken together, these findings demonstrate that AURKA inhibition suppresses the eIF2α-ATF4-ASNS pathway and induces apoptosis, with these effects being amplified in KEAP1-deficient cells.

### AURKA interacts with GCN2 and eIF2α to regulate their phosphorylation

**Fig. 5:**
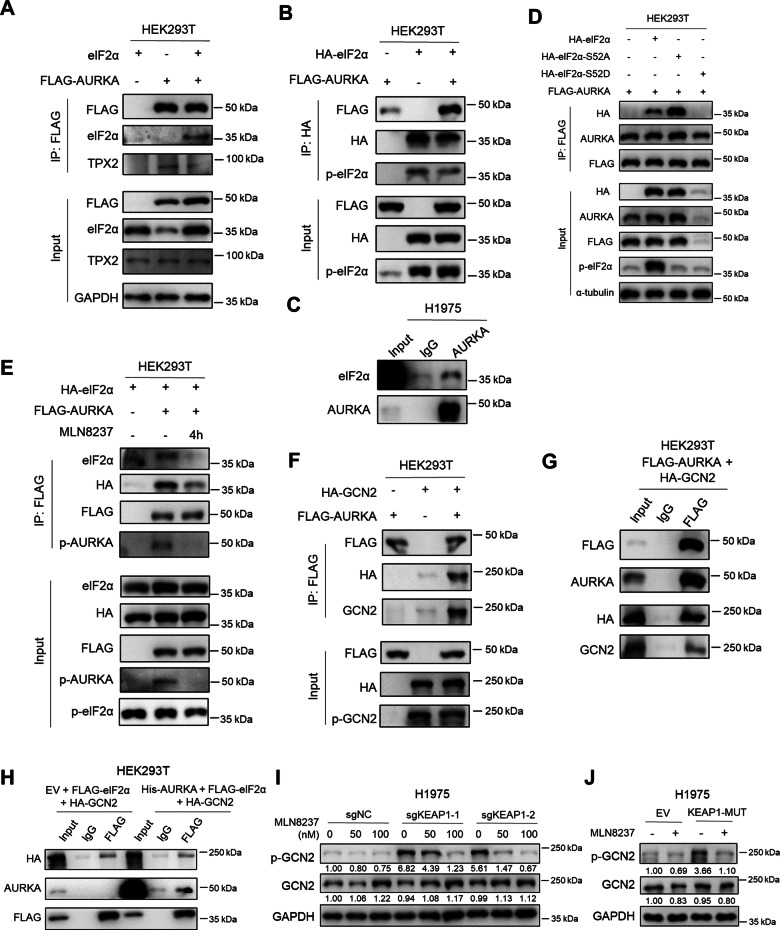
AURKA interacts with GCN2 and eIF2α to regulate their phosphorylation. **A** Co-IP assay using the FLAG antibody in HEK293T cells overexpressing eIF2α, FLAG-AURKA or both. **B** Co-IP assay using the HA antibody in HEK293T cells overexpressing HA-eIF2α, FLAG-AURKA or both. **C** Co-IP assay using the IgG or AURKA antibody in H1975 cells. **D** Co-IP assay using the FLAG antibody in HEK293T cells overexpressing FLAG-AURKA with control vector, HA-eIF2α, HA-eIF2α-S52A or HA-eIF2α-S52D. **E** Co-IP assay using the FLAG antibody in HEK293T cells overexpressing HA-eIF2α with control vector or FLAG-AURKA. The cells were treated with vehicle or 200 nM MLN8237 for 4 h before harvest. **F** Co-IP assay using the FLAG antibody in HEK293T cells overexpressing HA-GCN2, FLAG-AURKA or both. **G** Co-IP assay using the IgG or FLAG antibody in HEK293T cells overexpressing FLAG-AURKA and HA-GCN2. **H** Co-IP assay using the IgG or FLAG antibody in HEK293T cells overexpressing FLAG-eIF2α and HA-GCN2 with control vector or His-AURKA. **I** Western blot analysis in H1975-sgNC or sgKEAP1 cells (5 × 10^4^ cells/well) treated with 50 nM or 100 nM MLN8237 or vehicle for 72 h in 12-well plate. **J** Western blot analysis in H1975 cells overexpressing empty vector (EV) or mutant KEAP1 (KEAP1-MUT) (5 × 10^4^ cells/well) treated with 100 nM MLN8237 or vehicle for 72 h in 12-well plate. All the experiments were performed in three independent replicates.

Given that the downregulation of ASNS expression by AURKA inhibition is largely dependent on its suppressive effect on eIF2α phosphorylation, we further focused on the relationship between AURKA and eIF2α. Co-immunoprecipitation (co-IP) experiments were conducted in HEK293T cells transfected with eIF2α and FLAG-tagged AURKA vectors, revealing their binding interaction (Fig. 5A). This suggests that AURKA binds to eIF2α to regulate eIF2α-ATF4-ASNS pathway. Co-IP assays in MDA-MB-231 cells overexpressing FLAG-tagged AURKA also confirmed this interaction (Fig. S4A). We also performed co-IP assays using an HA antibody in HEK293T cells transfected with HA-tagged eIF2α and FLAG-tagged AURKA. In this result, we also observed the interaction of AURKA and eIF2α, but AURKA overexpression seemed to have no significant effect on the phosphorylation of eIF2α (Fig. 5B).

To demonstrate their endogenous binding, co-IP assays were conducted in H1975 cells. The eIF2α antibody detected a more pronounced band in the precipitation of the AURKA antibody compared to the IgG antibody, providing further evidence for the endogenous binding of AURKA and eIF2α (Fig. 5C). As eIF2α activity relies on phosphorylation at serine-52, we investigated whether their binding is correlated with eIF2α Ser52 phosphorylation. As anticipated, the inactive form of eIF2α with a Ser52-to-Alanine substitution (S52A) exhibited a stronger binding potential to AURKA compared to wildtype eIF2α (Fig. 5D). This indicates that AURKA tends to interact with eIF2α and promote its serine-52 phosphorylation when necessary. To determine whether the interaction between AURKA and eIF2α is dependent on the kinase activity of AURKA, co-IP assays were performed in HEK293T cells with or without MLN8237 treatment. The results showed that the binding between AURKA and eIF2α significantly weakened and the phosphorylation of eIF2α was also slightly downregulated after 4 h of MLN8237 treatment (Fig. 5E).

To determine whether AURKA directly phosphorylates eIF2α at serine-52, we conducted an in vitro kinase assay using GST-eIF2α and GST-AURKA recombinant proteins. The results revealed a weak background phosphorylation of eIF2α, and no stronger signal was observed in the group with both GST-eIF2α and GST-AURKA compared to the group with only GST-eIF2α (Fig. S4B). This finding suggests that AURKA may regulate eIF2α phosphorylation by interacting with a known kinase of eIF2α. Previous studies have implicated GCN2 as a kinase activated under conditions of amino acid starvation, leading to phosphorylation of eIF2α and induction of ATF4 and ASNS expression [47]. Co-immunoprecipitation assays demonstrated an interaction between AURKA and GCN2. Notably, AURKA overexpression did not significantly affect GCN2 phosphorylation or the interaction between GCN2 and eIF2α (Fig. 5F-H). However, p-GCN2 exhibited a marked increase in both KEAP1-knockdown cells and mutant KEAP1-overexpressing cells compared to KEAP1 wildtype cells. Importantly, this upregulation of p-GCN2 was significantly downregulated by treatment with the AURKA inhibitor MLN8237 in these cells (Fig. 5I, J), suggesting that AURKA may interact with GCN2 to sustain its phosphorylation. Besides, we observed that AURKA inhibition could also suppress the GCN2-eIF2α-ATF4-ASNS pathway induced by L-asparaginase treatment (Fig. S4C), but had no effect on ER stress activator tunicamycin-induced ASNS upregulation (Fig. S4D).

In summary, these findings demonstrate that AURKA may interact with GCN2 and eIF2α to maintain the phosphorylation of GCN2 and regulate the eIF2α-ATF4-ASNS pathway.

### Dependence of KEAP1-deficient NSCLC cells on ASNS determines their increased sensitivity to AURKA inhibition

**Fig. 6:**
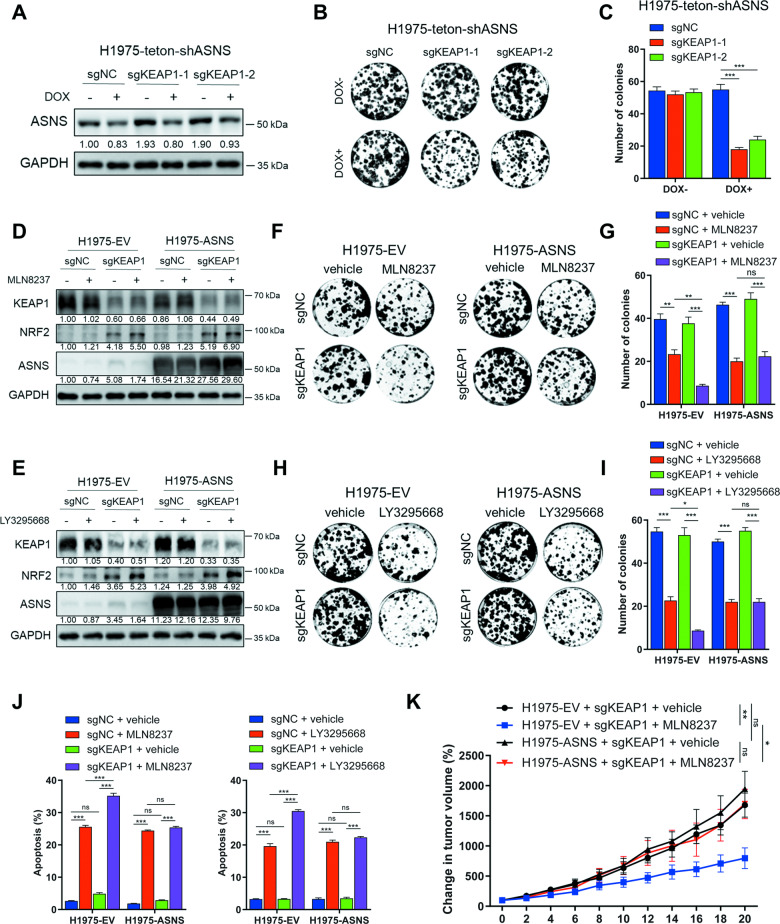
Dependence of KEAP1-deficient NSCLC cells on ASNS determines their increased sensitivity to AURKA inhibition. **A** Western blot analysis detecting the ASNS protein level in H1975 stable cell lines expressing a doxycycline (DOX)-induced shASNS (H1975-Teton-shASNS) with or without KEAP1 knockdown. The cells (5 × 10^4^ cells/well) were treated with vehicle or 2 μg/ml DOX for 72 h in 12-well plate. **B**, **C** Colony formation assays showing the cell viability in **A**. The cells (500 cells/well) were treated with vehicle or 2 μg/ml DOX. The representative pictures of colonies are showed in **B**, and the number of colonies are counted in **C**. **D**, **E** Western blot analysis in H1975 stable cell lines overexpressing empty vector (EV) or ASNS with or without KEAP1 knockdown. The cells (5 × 10^4^ cells/well) were treated with vehicle or 100 nM MLN8237 (**D**) or 100 nM LY3295668 (**E**) for 72 h in 12-well plate. **F-I** Colony formation assays showing the cell viability in **D** (**F**, **G**) and **E** (**H**, **I**). The representative pictures of colonies are showed in **F** and **H**, and the number of colonies are counted in **G** and **I**. The cells (500 cells/well) were treated with vehicle or 25 nM MLN8237 (**F**, **G**) or 37.5 nM LY3295668 (**H**, **I**). **J** The histogram showing the percentage of apoptosis cells in H1975-sgNC or sgKEAP1 cells (5 × 10^4^ cells/well) treated with vehicle or 100 nM MLN8237 (left) or 100 nM LY3295668 (right) for 72 h in 12-well plate. **K** The change in tumor volume after treatment with MLN8237 (30 mg/kg) or vehicle for the indicated time. Student t-tests were conducted on day 20 between the MLN8237 and vehicle treatment groups of H1975 cells overexpressing empty vector or ASNS with KEAP1 knockdown. In **A-J**, the experiments were performed in three independent replicates. Statistics, significance: one-way ANOVA (alpha=0.05) with Bonferroni correction (**C**, **G**, **I**, **J**); significance: two-tailed unpaired t-test (K); Error bars, SEM; ns not significant; *P < 0.05; **P < 0.01; ***P < 0.001.

According to previous reports, cells with mutant KEAP1 exhibit a heightened reliance on the uptake of exogenous non-essential amino acids, such as asparagine, due to increased glutamate consumption for glutathione (GSH) synthesis and the export of glutamate outside the cells [48]. To investigate whether the proliferation of KEAP1-deficient cells is more dependent on the expression of asparagine synthetase (ASNS) compared to KEAP1 wildtype cells, we generated a stable H1975 cell line that expresses a doxycycline (DOX)-inducible shASNS construct. Subsequently, we introduced the knockdown or mutant KEAP1 plasmids in this cell line. Colony formation assays were performed to assess the cell growth ability. The findings revealed a significant inhibition of growth in KEAP1-knockdown cells and mutant KEAP1-overexpressing cells when ASNS expression was downregulated through DOX treatment. In contrast, the proliferation of KEAP1 wildtype cells seemed unaffected by DOX treatment (Fig. 6A-C and Fig. S5A-C). These results suggested the dependence of KEAP1-deficient NSCLC cells on ASNS expression. Furthermore, the growth inhibition caused by ASNS downregulation could be rescued by a higher dose of L-asparagine supplementation in KEAP1-knockdown cells (Fig. S5D, E).

To further confirm that the enhanced growth inhibition effect of AURKA inhibitors in KEAP1-deficient cells depends on the downregulation of ASNS expression, we stably overexpressed ASNS in H1975 cells prior to KEAP1 knockdown. The cells were then treated with AURKA inhibitors MLN8237 or LY3295668, and the overexpression of ASNS and knockdown of KEAP1 were confirmed through western blot analysis (Fig. 6D, E). Subsequently, colony formation assays were conducted, and it was observed that the increased sensitivity to AURKA inhibitors (MLN8237 and LY3295668) resulting from KEAP1 knockdown was abolished by ASNS overexpression in H1975 cells (Fig. 6F-I). Consistently, the increased proportion of apoptotic cells observed in KEAP1-knockdown cells treated with AURKA inhibitors was significantly reduced by ASNS overexpression (Fig. 6J). We also observed the similar rescue effect of ASNS overexpression on AURKA inhibition in mutant KEAP1-overexpressing H1975 cells (Fig. S5F-H). Finally, the subcutaneous tumor formation experiments in nude mice were conducted using the H1975 KEAP1-knockdown cells with or without ASNS overexpression treated with vehicle or MLN8237. The results showed that ASNS overexpression nearly abolished the effect of MLN8237 in sgKEAP1 cells in vivo (Fig. 6K). Collectively, these results demonstrate that the dependency of KEAP1-deficient NSCLC cells on ASNS expression determines their heightened sensitivity to AURKA inhibitors.

## Discussion

**Fig. 7:**
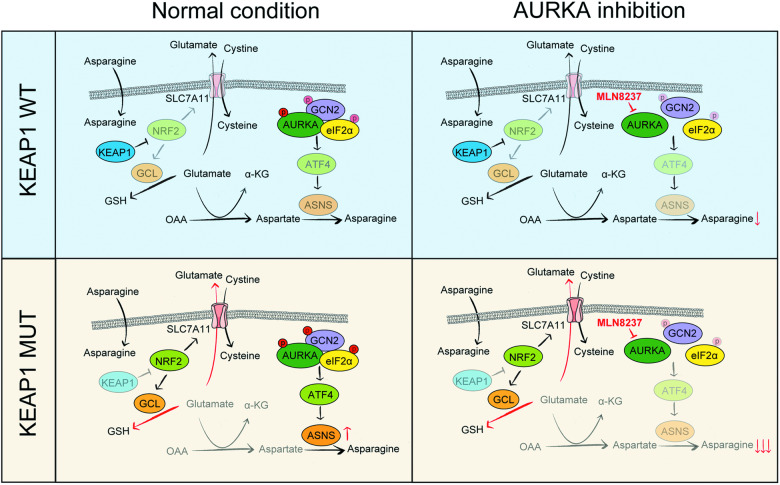
Working model depicting the mechanism that KEAP1 deficiency sensitizes NSCLC cells to AURKA inhibition. In KEAP1 mutant (MUT) tumor cells, the NRF2 pathway is activated, leading to the increase in the consumption of glutamate for GSH synthesis and the export of glutamate outside the cell through SLC7A11 in exchange for cystine, compared with KEAP1 wildtype (WT) cells. Reduced intercellular glutamate level results in activation of GCN2 and eIF2α phosphorylation and ATF4-mediated ASNS expression in KEAP1 mutant tumor cells. AURKA interacts with GCN2 and eIF2α, and AURKA inhibition using its kinase inhibitors such as MLN8237 can remarkably suppress the activated GCN2-eIF2α-ATF4-ASNS axis in KEAP1 mutant tumor cells, resulting in a significant decrease in asparagine levels, increased apoptosis, and further inhibition of cell proliferation.

In this study, we found that the highly prevalent KEAP1 deficiency sensitizes NSCLC cells to AURKA inhibitors, establishing AURKA as a potential therapeutic target in specific lung cancer. We elucidated the underlying mechanism by revealing the involvement of the GCN2-eIF2α-ATF4-ASNS axis, which is activated in KEAP1-deficient cells. Notably, we discovered that AURKA interacts with GCN2 and eIF2α, and that AURKA inhibitors can downregulate ATF4-activated ASNS expression by inhibiting GCN2 and eIF2α phosphorylation (Fig. 7). These results highlight the vulnerability of AURKA in KEAP1 mutant NSCLC.

KEAP1 mutant NSCLC exhibits resistance to conventional therapeutic approaches, including chemotherapy, radiotherapy, targeted therapy, and immunotherapy [39]. We also confirmed that KEAP1-deficient cells were evidently resistant to the MEK inhibitor MEK162 and the chemotherapy drug CPT (Fig. S2G). Current investigations are focusing on compounds such as the MTOR inhibitor TAK-228 and the glutaminase inhibitor CB-839, which target the mTOR pathway and glutaminolysis, essential for the survival of KEAP1 or NRF2 mutant tumors [36, 49–52]. In this study, we identified AURKA inhibitors, including MLN8237, which are undergoing clinical trials in various tumor types, including NSCLC, as potential targeted drugs for KEAP1 mutant NSCLC. Notably, AURKA inhibitors regulate amino acid biosynthesis, making them attractive options for treating KEAP1 mutant NSCLC by targeting both metabolism and cell proliferation. Given that canonical metabolic drugs may also harm normal cells, the combination of AURKA inhibitors with L-asparaginase, as reported in a recent study for KEAP1 mutant lung cancer [48], holds promise for achieving enhanced therapeutic efficacy and merits further investigation.

Previous studies have identified specific mutation types associated with tumor sensitivity to AURKA inhibitors, such as RB1 deficiency [53] or loss of SMARCA4 or ARID1A [54, 55]. However, given the diverse mutation patterns observed in different tumor types, unbiased screens using multiple CRISPR/Cas9 libraries in additional tumor types are necessary to comprehensively explore indications for AURKA inhibitors. This research provides valuable insights into expanding the scope of tumor sensitivity to AURKA inhibitors and highlights the need for future studies in diverse tumor types.

Tumor metabolism plays a pivotal role in various aspects of tumor development, including cell proliferation, stress response, and drug resistance [56, 57]. Previous studies have reported the involvement of AURKA in tumor metabolism. Notably, AURKA has been implicated in phosphorylating LKB1, resulting in the suppression of the LKB1/AMPK signaling pathway and promoting the proliferation, invasion, and migration of non-small cell lung cancer cells [6]. Furthermore, AURKA has been associated with the regulation of glycolysis and fatty acid oxidation [21, 22]. Our study provides novel insights into the regulatory role and mechanism of AURKA in amino acid synthesis, expanding its involvement in metabolism. Importantly, our findings highlight the potential therapeutic applications of AURKA inhibitors in metabolism-related diseases. While AURKA is well-known as a mitotic kinase, further investigation is warranted to determine its potential role in activating the phosphorylation of eIF2α and its impact on the biosynthesis of specific amino acids, such as serine, glycine, or asparagine, during mitosis. Future studies in this area will enhance our understanding of AURKA’s multifaceted functions in tumor metabolism.

Previous studies have demonstrated the role of ASNS in regulating the proliferation and metastasis of various cancers, including lung cancer, breast cancer, colorectal cancer and glioma [58–62]. Significantly, recent insights have underscored the therapeutic promise of targeting ASNS in KRAS-mutant NSCLC, where oncogenic KRAS stimulates ASNS expression through the PI3K-AKT-NRF2-ATF4 axis [45]. ATF4 has over 200 described targets [63], and the NRF2-ATF4 pathway, are also linked to the activation of serine biosynthesis [25]. In our investigation, we identified ASNS as the most prominent target in the amino acid biosynthesis pathway, showing substantial downregulation upon AURKA inhibition. Moreover, we noted a pronounced upregulation of the eIF2α-ATF4-ASNS signaling pathway, coupled with a discernible reliance on ASNS in KEAP1-deficient NSCLC cells. The overexpression of ASNS successfully countered the impact of AURKA inhibition in these cells. Consequently, we posit that while the regulation of ASNS expression may be a crucial contributor, it might not stand alone as the sole mechanism underlying the heightened susceptibility of KEAP1-deficient cells to AURKA inhibition.

Interestingly, we also observed ATF4 transcriptional upregulation in KEAP1-deficient cells, likely attributed to NRF2 activation (Fig. S3C), which is consistent with previous reports. Nonetheless, our research suggests that the inhibition of AURKA does not significantly impact the levels of NRF2 protein and instead induces the upregulation of its conventional targets, including HMOX1 or GCLM (Fig. 4D and Fig. S3C). This implies that AURKA modulates the ATF4-ASNS axis mainly through p-eIF2α, while exhibiting no significant dependence on the NRF2 pathway.

In our study, we have validated the interaction between AURKA and eIF2α, a finding consistent with previous reports [64]. Significantly, our investigation demonstrates that AURKA inhibition results in the downregulation of p-eIF2α and its upstream regulator p-GCN2. Our results indicate that AURKA interacts with GCN2, although GCN2 phosphorylation is not modulated by AURKA overexpression. This aligns with the established knowledge that GCN2, a well-known eIF2α kinase, is activated by binding uncharged tRNAs under conditions of amino acid deficiency [65, 66]. Hence, AURKA may interact with GCN2 and sustain its phosphorylation only after GCN2 activation. Despite the absence of a significant change in p-AURKA, our findings show a substantial upregulation of p-GCN2 in KEAP1-deficient cells compared to KEAP1 wildtype cells. Previous studies have suggested that KEAP1 loss enhances the reliance on exogenous non-essential amino acids (NEAA), such as asparagine [48]. We hypothesize that in KEAP1-deficient cells, there is an increase not only in the consumption of exogenous NEAA but also in the activation of the endogenous amino acid synthesis pathway through the GCN2-eIF2α-ATF4 axis. This activation supports cell survival and growth, accompanied by heightened antioxidant production.

In conclusion, our study demonstrates that AURKA interacts with GCN2 and eIF2α to regulate asparagine synthesis in KEAP1-deficient NSCLC. AURKA inhibitors hold promise as therapeutic drugs for KEAP1-deficient NSCLC.

## Materials and methods

### Cell culture, transfection and transduction

The NSCLC cell lines H1975, H3122, H1650, H2122 and HCC44 were cultured in RPMI 1640 (GIBCO) supplemented with 10% (v/v) fetal bovine serum (GIBCO). The HEK293T cells and the breast cancer cell line MD-MBA-231 were cultured in Dulbecco’s modified Eagle medium (GIBCO) supplemented with 10% (v/v) fetal bovine serum (GIBCO). All cell lines were validated to be mycoplasma-free and cultured in a 5% CO_2_ atmosphere at 37 °C. Transfection of plasmids in HEK293T cells was performed using PEI (Sigma) according to the manufacturer’s instructions. To establish stable cell lines, lentivirus was produced following the procedures we previously used [16]. Cells were transduced with lentivirus in the presence of 8 mg/ml Polybrene (Sigma-Aldrich, sc-134220). After 48 h, the corresponding antibiotic was added to select transduced cells.

### Plasmid construction

For transient overexpression of AURKA, eIF2α or GCN2, the CDS sequences were cloned into pcDNA3.1, pcDNA3.1-3×FLAG or pcDNA3.1-HA plasmids using the ClonExpress II One Step Cloning Kit (Vazyme). For stable overexpression of AURKA, the CDS sequence was cloned into plvx-3×FLAG with puromycin resistance. For stable overexpression of KEAP1-MUT, the CDS sequence was amplified from the RNA of HCC44 cells and cloned into plvx-PURO plasmid with puromycin resistance. For stable overexpression of ASNS, the CDS sequence was cloned into a plvx-IRES-BSD plasmid with blasticidin resistance. For knockdown of KEAP1 or NRF2, the sgRNA oligos were inserted into the lentiCRISPR v2 plasmid (Addgene #52961). For inducible knockdown of ASNS, the shRNA oligos were inserted into the Teton-pLKO-BSD plasmid with blasticidin resistance. All plasmids were verified by sequencing. The primers for plasmid construction are listed in Supplementary Table 5.

### CRISPR/Cas9 metabolic library screen and sequencing

The CRISPR/Cas9 library screens were performed as described previously [16]. Briefly, the metabolic library (Human CRISPR Metabolic Gene Knockout Library, Addgene #110066) was packaged into lentivirus with pMD2.G and psPAX2 plasmids using PEI (Sigma) in HEK293T cells. The metabolic library contains ~30000 Lenti-v1 plasmids targeting 2981 metabolism-related genes (10 sgRNAs for each gene and 500 intergenic control sgRNAs) [40]. After 48 h transfection, the metabolic library lentivirus was collected and infected into MDA-MB-231 or H1975 cells with an MOI of 0.3 for 48 h followed by puromycin selection for 7 days. When cells reached confluency, they were passaged by mixing all cells from each culture dish and re-seeded into new dishes. Subsequently, cells were divided into vehicle (DMSO) and MLN8237 (150 nM) treatment groups in MDA-MB-231 cells, and vehicle (DMSO) and MLN8237 (50 nM) treatment groups in H1975 cells. And cells were collected after 3 d and 7 d of drug treatment for MDA-MB-231 cells, and 5 d and 7 d for H1975 cells, respectively. For each group, the number of collected cells were guaranteed to be over 2 × 10^7^ to achieve 300 × coverage. Genomic DNA was extracted using an HP Tissue DNA Midi Kit (D5197, OMEGA). The sgRNAs were amplified by two-round PCR using primers to construct libraries. The libraries were then sequenced on an Illumina HiSeq2000. The sequencing data were processed and analyzed using the MAGeCK-MLE software package (version 0.5.9.5). The primers used for library construction were listed in Supplementary Table 5.

### RNA Extraction and qRT-PCR

RNA was extracted with the HiPure Total RNA Plus Mini Kit (Magen). cDNA was synthesized using a HiScript III All-in-one RT SuperMix Perfect for qPCR kit (Vazyme) according to the manufacturer’s protocol. QPCR assays were performed in triplicates with the ChamQ Universal SYBR qPCR Master Mix (Vazyme). The relative quantitation (RQ) of gene expression was normalized to GAPDH. The qRT-PCR primers are listed in Supplementary Table 5.

### RNA sequencing (RNA-seq)

For RNA-seq, MDA-MB-231 cells were treated with AURKA inhibitors (150 nM MLN8237) for 72 h or with AURKA depletion (AURKA KO). The library was constructed and sequenced by Novogene with Illumina HiSeq2000.

### Colony formation assay

For colony formation assays, 500 to 1000 cells were plated into 12-well plates and then treated with candidate drugs. The medium and drugs were renewed every 4 days. After 8 to 10 days, the colonies were fixed using methanol for 20 min and stained with a crystal violet solution for 20 min at room temperature. The plates were imaged using the ChemiDoc MP Imaging System (Bio-Rad).

### Cell viability assay

For cell viability assays, 1 × 10^3^ cells were plated into 96-well plates. After 24 h, the culture medium was replaced with fresh medium containing the indicated concentrations of inhibitors for 72 h. Then, the number of viable cells was evaluated using the Cell Counting Kit 8 (CCK-8, 40203ES92, Yeasen) according to the manufacturer’s instructions. The absorbance at 450 nm reflecting live cell numbers was measured and the relative viability (%) of all groups was normalized to the vehicle group.

### Apoptosis analysis

For apoptosis analyses, cells were treated with the candidate drugs for 72 h. The attached cells were trypsinized and collected with the dead cells in the supernatant. All cells were washed with PBS twice and stained with Annexin V-FITC/PI using the Annexin V-AF647 Apoptosis Detection Kit (ES Science, AP006) for 30 min according to the manufacturer’s instructions. Then, the stained cells were evaluated by flow cytometry using the CytoFLEX platform (Beckman Coulter).

### Western blot

Total proteins were extracted from cells using the RIPA buffer supplemented with protease inhibitors and phosphatase inhibitors. The protein concentration was determined using Coomassie brilliant blue G250 dye at an absorbance of 595 nm on a microplate reader. Then, a certain amount of protein samples with loading buffer was denatured at 100 °C for 10 min and separated by SDS-PAGE and then transferred to 0.2 μm PVDF membranes (Merck Millipore). Primary antibodies used were as follows: rabbit anti-phosphorylated AURKA/AURKB/AURKC (2914 S, CST), rabbit anti-AURKA (14475 S, CST), mouse anti-AURKA (A1231, Sigma), mouse anti-GAPDH (60004-1-Ig, Proteintech), mouse anti-phosphorylated eIF2α (68023-1-Ig, Proteintech), rabbit anti-eIF2α (11170-1-AP, Proteintech), rabbit anti-ATF4 (10835-1-AP, Proteintech), rabbit anti-NRF2 (16396-1-AP, Proteintech), rabbit anti-KEAP1(10503-2-AP, Proteintech), rabbit anti-ASNS (14681-1-AP, Proteintech), mouse anti-FLAG (F1804, Sigma), rabbit anti-HA (51064-2-AP, Proteintech), rabbit anti-HA (3724 S, CST), mouse anti-β-Actin (66009-1-Ig, Proteintech), mouse anti-α-tubulin (66031-1-Ig, Proteintech), rabbit anti-GCN2 (A2307, Abclonal), rabbit anti-phosphorylated GCN2 (ab75836, Abcam), rabbit anti-TPX2 (12245 S, CST). The western blots were quantified using Image J software.

### Co-Immunoprecipitation (Co-IP)

The cells were harvested and lysed in NETN buffer (20 mM Tris-HCl, pH 8.0; 100 mM NaCl; 0.5% NP-40; and 1 mM EDTA) supplemented with protease inhibitors and phosphatase inhibitors on ice for 30 min. Then, the cell lysates were centrifugated at 15,000 × g at 4 °C for 10 min. The supernatants were incubated with 1 μg antibody overnight at 4 °C while rotating. The antibodies used for co-IP were as follows: mouse anti-FLAG (F1804, Sigma), rabbit anti-HA (51064-2-AP,Proteintech), and mouse anti-AURKA (A1231, Sigma) antibodies. Subsequently, the precipitations were incubated with 30 μl Protein A/G Magnetic Beads (HY-K0202, MCE) at 4 °C for 2 h. After incubation, the beads were washed five times with the NETN buffer and finally eluted by directly boiling in loading buffer. Finally, the samples were analyzed by western blot.

### In vitro kinase assay

Recombinant human protein GST-eIF2α (0.5 μg, Proteintech) was incubated with GST-AURKA (0.5 μg) in kinase buffer supplemented with 200 μM ATP in a final volume of 30 μl for 30 min at 30 °C. The reaction was terminated by the addition of loading buffer. Samples were analyzed by SDS-PAGE and the phosphorylated eIF2α was detected using anti-phosphorylated eIF2α (phospho-Ser52) antibody.

### Mouse experiments

The cells of H1975 stable cell lines were harvested, suspended in culture medium and then counted. The cell suspension was mixed with matrigel (354234, Corning) at a 7:3 ratio and the cell concentration was adjusted to 2 × 10^7^ cells/ml. Then, 100 μl cells were inoculated subcutaneously in the flank of 4-week-old female nude mice. Once the tumors reached ~50 mm^3^, the mice were randomly divided into four groups (six mice per group) and treated with intraperitoneal injection of MLN8237 (30 mg/kg) or vehicle (DMSO) once daily. The length and width of each tumor were measured using calipers and the mice were weighed every 2 to 3 days after treatment initiation. The tumor volume was calculated using the equation V = (length × width^2^) / 2. On day 21, the mice were euthanized, and the tumor xenografts were immediately dissected and weighed using an electronic balance. Statistical analysis and significance testing were conducted using GraphPad Prism 8.

### Bioinformatics analysis

For RNA-seq analysis, the raw sequencing data were qualified using FastQC and the differentially expressed genes (DEG) were calculated using the DESeq2 package. 0 < *p* < 0.05 and log2 (Fold Change) < −0.5 were set as the thresholds for significantly downregulated genes in Supplementary Table 3. The overlapped downregulated genes between AURKA inhibition and knockout group in Supplementary Table 4 were employed to the KEGG pathway analysis using the DAVID database (https://david.ncifcrf.gov/). For the co-expression patterns between AURKA and ASNS, the TCGA RNA-seq data of LUAD and LUSC was analyzed using starBase database (https://rnasysu.com/encori/). For drug sensitivity analysis, the IC50 values of the AURKA inhibitor alisertib in 82 NSCLC cell lines was downloaded from the Cancer Drug Sensitivity Genomics database GDSC (https://www.cancerrxgene.org/). The cell line mutation information of KEAP1 was queried through the COSMIC database (http://cancer.sanger.ac.uk/cosmic/) and showed in Supplementary Table 2.

### Statistical analysis

The results were presented as mean ± S.E.M unless otherwise noted. Unpaired Student’s t-test was used for comparisons between the two groups. One-Way ANOVA was used for comparisons between two groups among multiple groups. Statistical analyses were performed with GraphPad Prism 8. The significance *P* values were denoted by asterisks as follows: *P < 0.05, **P < 0.01 and ***P < 0.001.

### Supplementary information

Supplementary Figures

checklist

Supplementary Table 1

Supplementary Table 2

Supplementary Table 3

Supplementary Table 4

Supplementary Table 5

Original Data File

## Data Availability

The data generated during and/or analyzed during the current study are available from the corresponding author on request.
