# Supplementary material for: AURKA emerges as a vulnerable target for KEAP1-deficient non-small cell lung cancer by activation of asparagine synthesis
Source: Cell Death Dis. 2024 Mar 23;15(3):233. doi: 10.1038/s41419-024-06577-x (PMC10960834; doi:10.1038/s41419-024-06577-x)
Supplement: Supplementary file 1 — Supplementary Figures [file 41419_2024_6577_MOESM1_ESM.docx]

**Supplementary Figures**


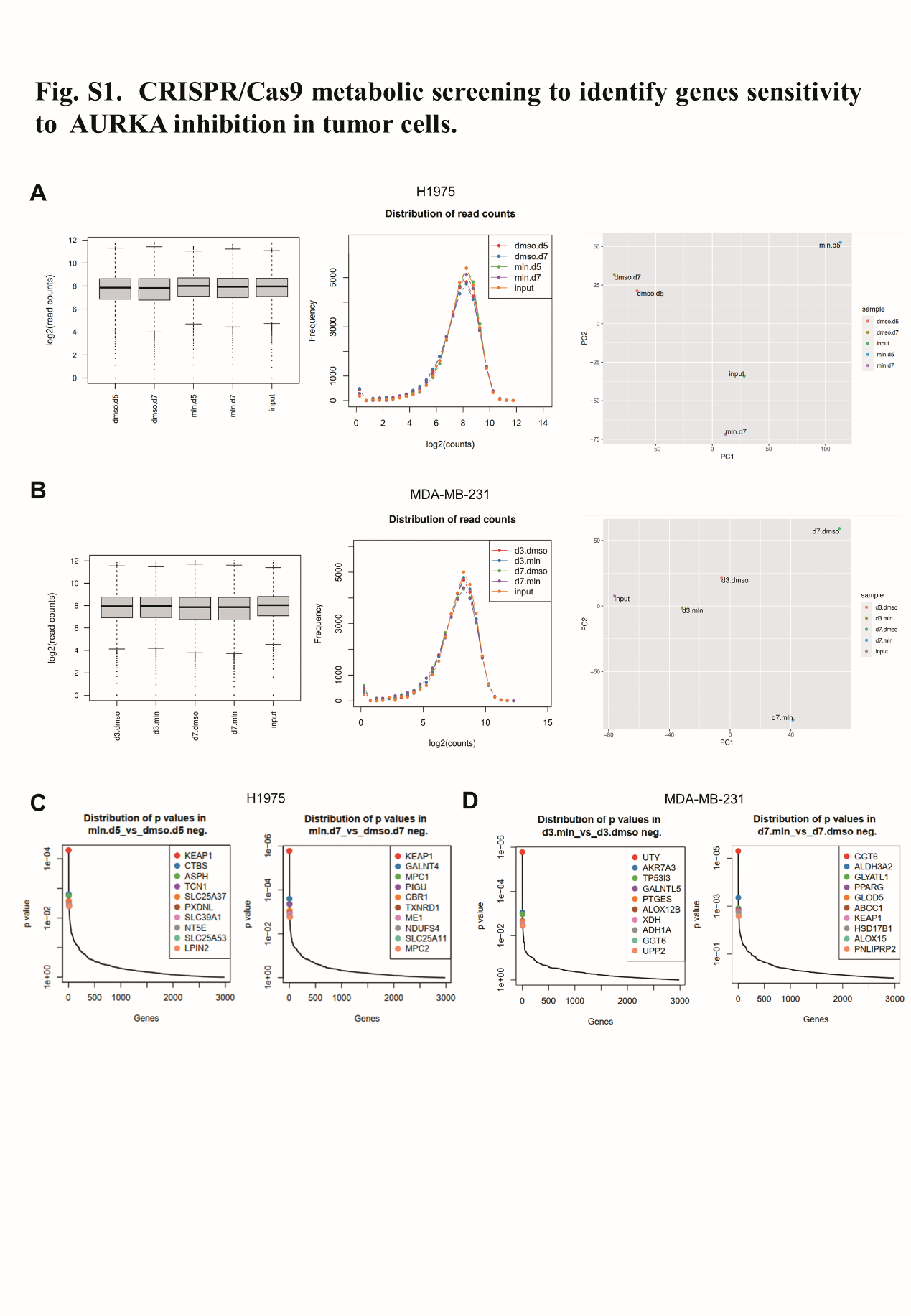


**Fig. S1. CRISPR/Cas9 metabolic screens to identify genes sensitizing tumor cells to AURKA inhibitors.**

**A, B** The histogram of median normalized read counts (left), the distribution of read counts in each group groups (middle) and the distinct evolutionary routes over time (right) in vehicle or MLN8237-treated groups in H1975 cells (**A**) and MDA-MB-231 cells (**B**). **C, D** Distribution of p value of the negatively selected genes in MLN8237-treated H1975 (**C**) or MDA-MB-231 (**D**) cells compared with the vehicle-treated cells at day 5 (left) and day 7 (right) (**C**) or day 3 (left) and day 7 (right) (**D**).


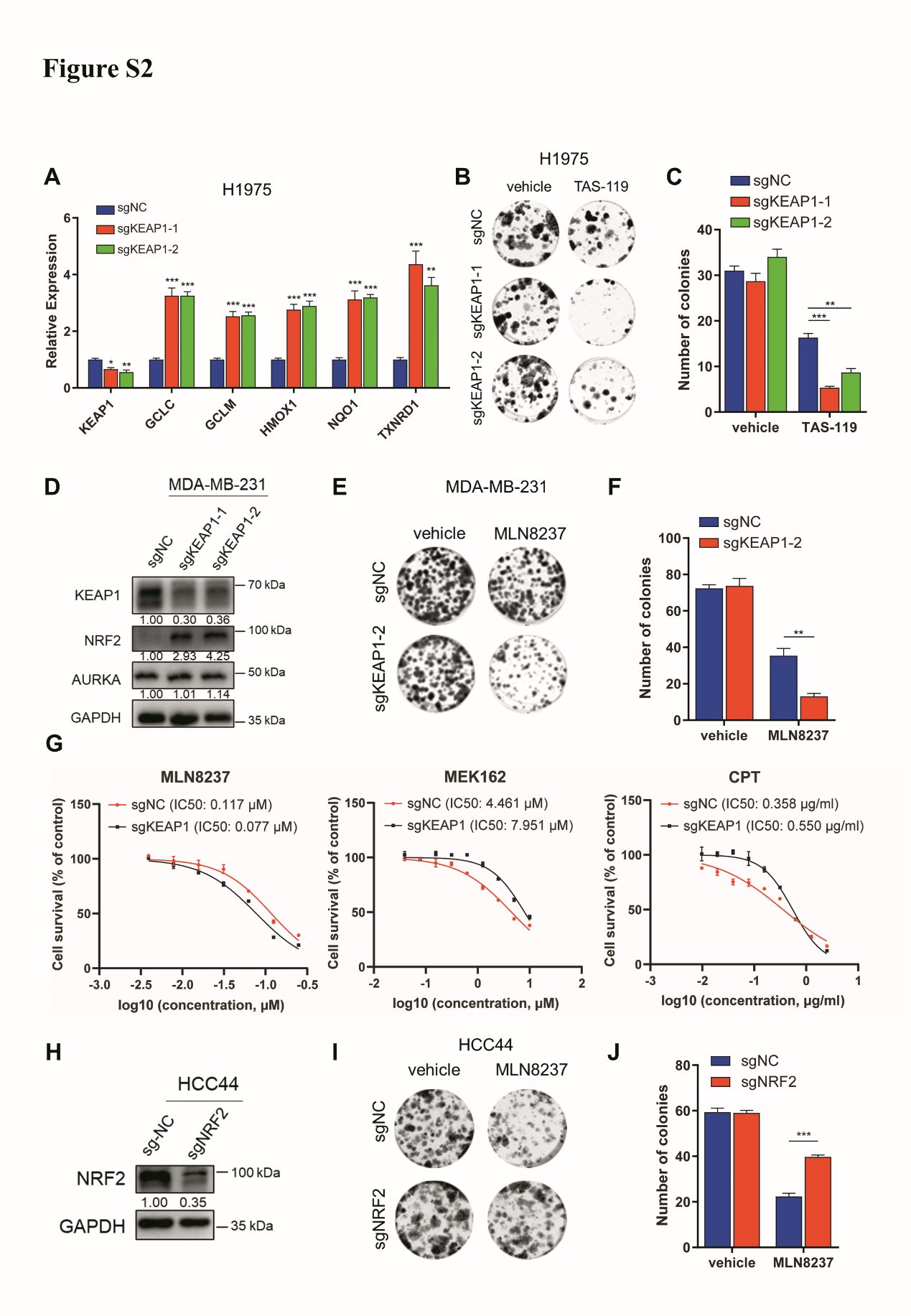


**Fig. S2. KEAP1-NRF2 pathway regulates the sensitivity of the cells to AURKA inhibition.**

**A** QPCR analysis showing the relative expression of the indicated genes in H1975 cells with or without KEAP1 knockdown. **B, C** Colony formation assays showing the cell viability of H1975-sgNC and sgKEAP1 cells (500 cells/well) treated with vehicle or 25 nM TAS-119. The representative pictures of colonies are showed in **B**, and the number of colonies are counted in **C**. **D** Western blot analysis showing the knockdown effect of KEAP1 in MDA-MB-231 cells. **E, F** Colony formation assays showing the cell viability of MDA-MB-231-sgNC and sgKEAP1 cells (500 cells/well) treated with vehicle or 50 nM of MLN8237. The representative pictures of colonies are showed in **E**, and the number of colonies are counted in **F**. **G** The cell viability of MDA-MB-231 cells (1000 cells/well) treated with the indicated doses of drugs for 72 h. All groups were normalized to the vehicle group. **H** Western blot analysis showing the knockdown effect of NRF2 in HCC44 cells. **I, J** Colony formation assays showing the cell viability of HCC44-sgNC and sgNRF2 cells (350 cells/well) treated with vehicle or 50 nM MLN8237. The representative pictures of colonies are showed in **I**, and the number of colonies are counted in **J**. All the experiments were performed in three independent replicates. Statistics, significance: one-way ANOVA (alpha=0.05) with Bonferroni correction (**A, C**); significance: two-tailed unpaired t-test (**F, J**); Error bars, SEM; **P* < 0.05; ***P* < 0.01; ****P* < 0.001.


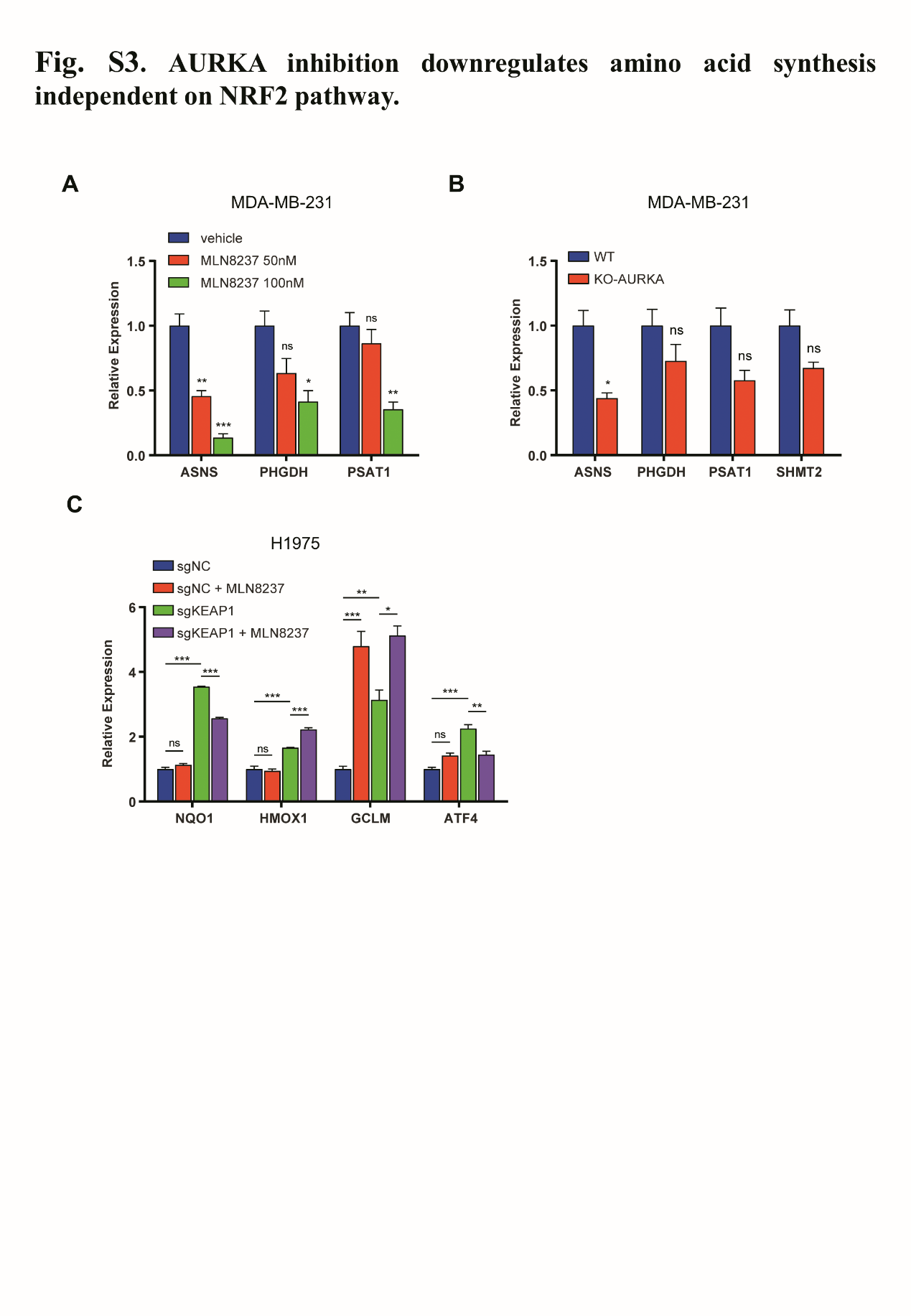


**Fig. S3. AURKA inhibition downregulates amino acid synthesis independent on NRF2 pathway.**

**A, B** The relative expression of the indicated genes of amino acid biosynthesis in MDA-MB-231 cells (5 × 10^4^ cells/well) with treatment of the indicated doses of MLN8237 (**A**) or AURKA depletion (**B**) in 12-well plate, determined by qPCR analysis. **C** The relative expression of the indicated genes determined by qPCR analysis in H1975-sgNC or sgKEAP1 cells (5 × 10^4^ cells/well) treated with 100 nM MLN8237 or vehicle for 72 h in 12-well plate. All the experiments were performed in three independent replicates. Statistics, significance: one-way ANOVA (alpha=0.05) with Bonferroni correction (**A, C**); significance: two-tailed unpaired t-test (**B**); Error bars, SEM; ns, not significant; **P* < 0.05; ***P* < 0.01; ****P* < 0.001.


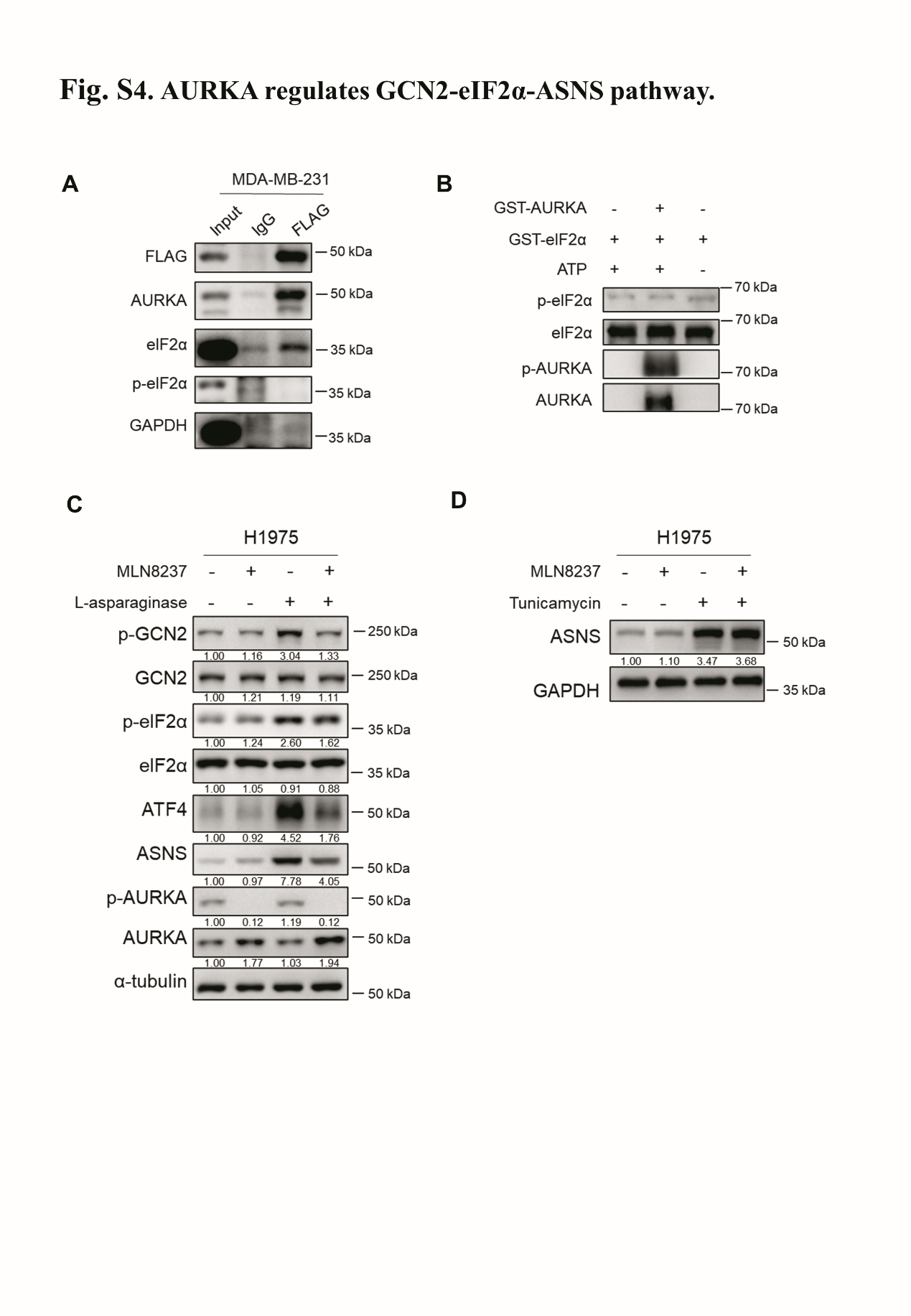


**Fig. S4. AURKA regulates GCN2-eIF2α-ASNS pathway.**

**A** Co-IP assay using the IgG or FLAG antibody in MDA-MB-231 cells overexpressing FLAG-AURKA. **B** In vitro kinase assay using GST-eIF2α with or without GST-AURKA. The phosphorylation of eIF2α and AURKA was detected using western blot. **C** Western blot analysis in H1975 cells (5 × 10^4^ cells/well) treated with 50 nM MLN8237, 0.1U/ml L-asparaginase or both for 72 h in 12-well plate. **D** Western blot analysis in H1975 cells (5 × 10^4^ cells/well) treated with 100 nM MLN8237, 0.2 ug/ml tunicamycin or both for 72 h in 12-well plate. All the experiments were performed in three independent replicates.


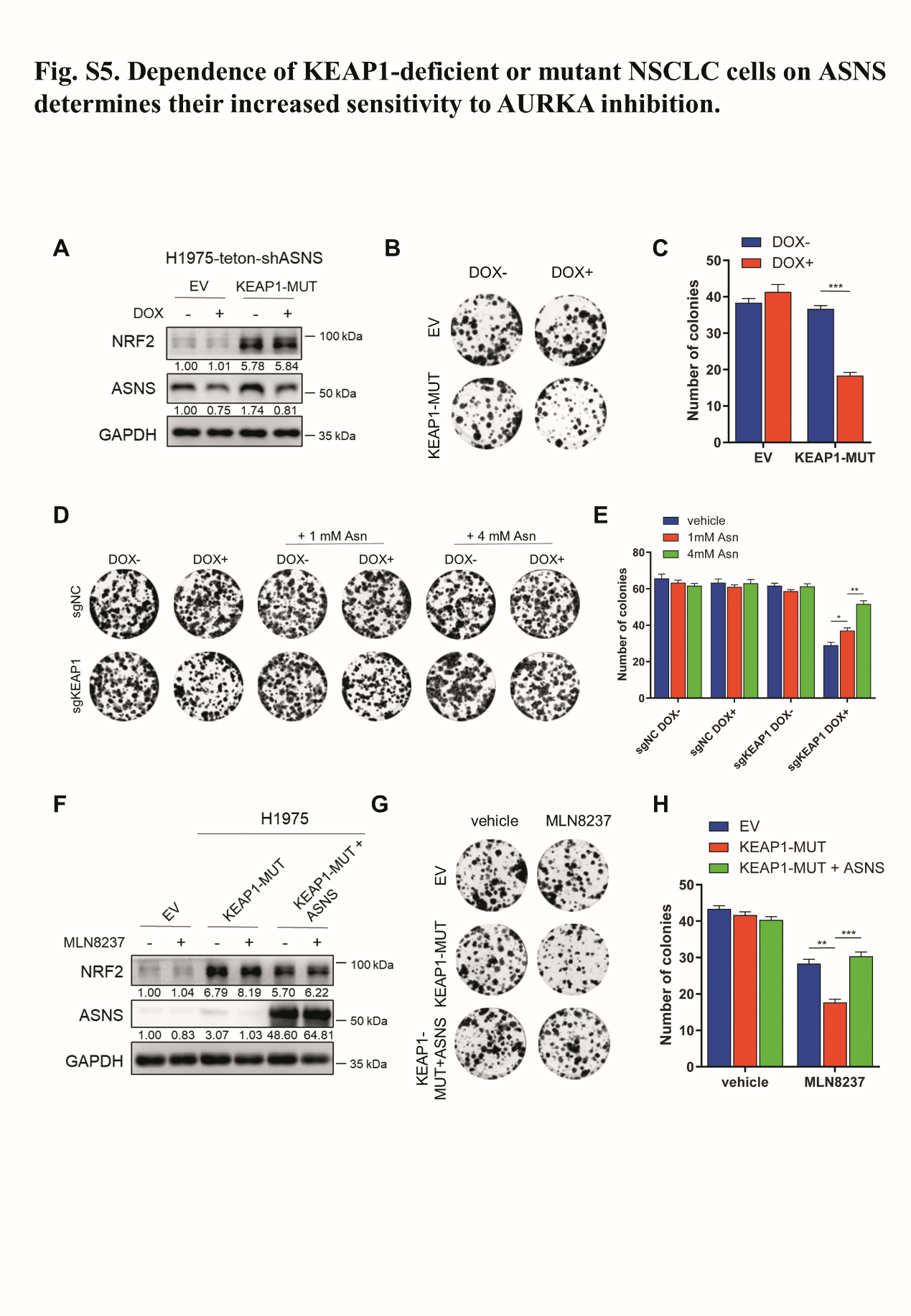


**Fig. S5. Dependence of KEAP1-deficient or mutant NSCLC cells on ASNS determines their increased sensitivity to AURKA inhibition.**

**A** Western blot analysis in doxycycline (DOX)-induced H1975-Teton-shASNS stable cell lines overexpressing empty vector (EV) or mutant KEAP1 (KEAP1-MUT). The cells (5 × 10^4^ cells/well) were treated with vehicle or 2 μg/ml DOX for 72 h. **B, C** Colony formation assays showing the cell viability in **A**. The cells (500 cells/well) were treated with vehicle or 2 μg/ml DOX. The representative pictures of colonies are showed in **B**, and the number of colonies are counted in **C**. **D, E** Colony formation assays showing the cell viability in H1975-teton-shASNS cells with or without KEAP1 knockdown. The cells (500 cells/well) were treated with vehicle or 2 μg/ml DOX and supplemented with indicated dose of L-asparagine in the medium. The representative pictures of colonies are showed in **D**, and the number of colonies are counted in **E**. **F** Western blot analysis in H1975 stable cell lines overexpressing empty vector (EV) or mutant KEAP1 (KEAP1-MUT) with or without ASNS overexpression. The cells (5 × 10^4^ cells/well) were treated with vehicle or 100 nM MLN8237 for 72 h. **G, H** Colony formation assays showing the cell viability in **F**. The cells (500 cells/well) were treated with vehicle or 25 nM MLN8237. The representative pictures of colonies are showed in **G**, and the number of colonies are counted in **H**. All the experiments were performed in three independent replicates. Statistics, significance: one-way ANOVA (alpha=0.05) with Bonferroni correction (**E, H**); significance: two-tailed unpaired t-test (**C**); Error bars, SEM; **P* < 0.05; ***P* < 0.01; ****P* < 0.001.
