# Supplementary material for: AURKA emerges as a vulnerable target for KEAP1-deficient non-small cell lung cancer by activation of asparagine synthesis
Source: Cell Death Dis. 2024 Mar 23;15(3):233. doi: 10.1038/s41419-024-06577-x (PMC10960834; doi:10.1038/s41419-024-06577-x)

Figure 2C

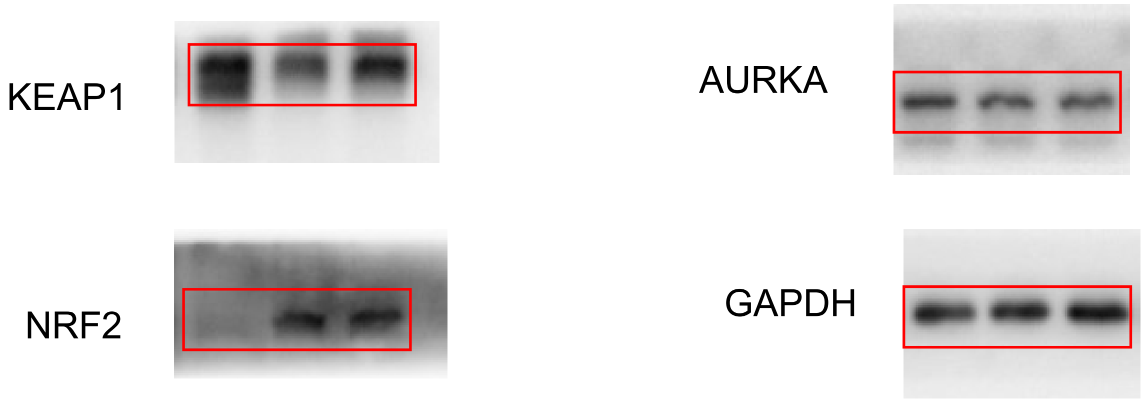

Figure 2D

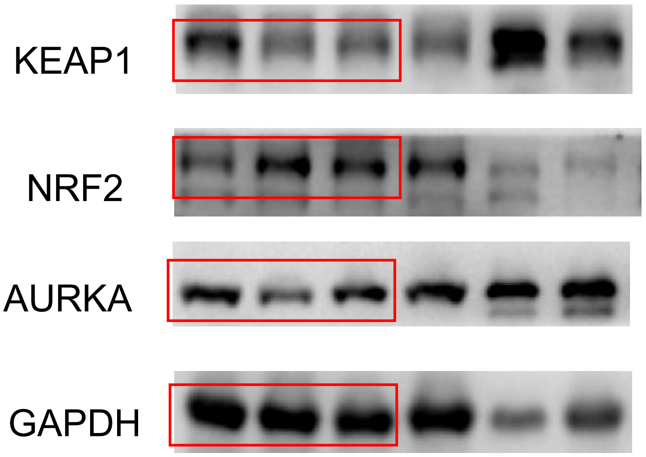

Figure 2I

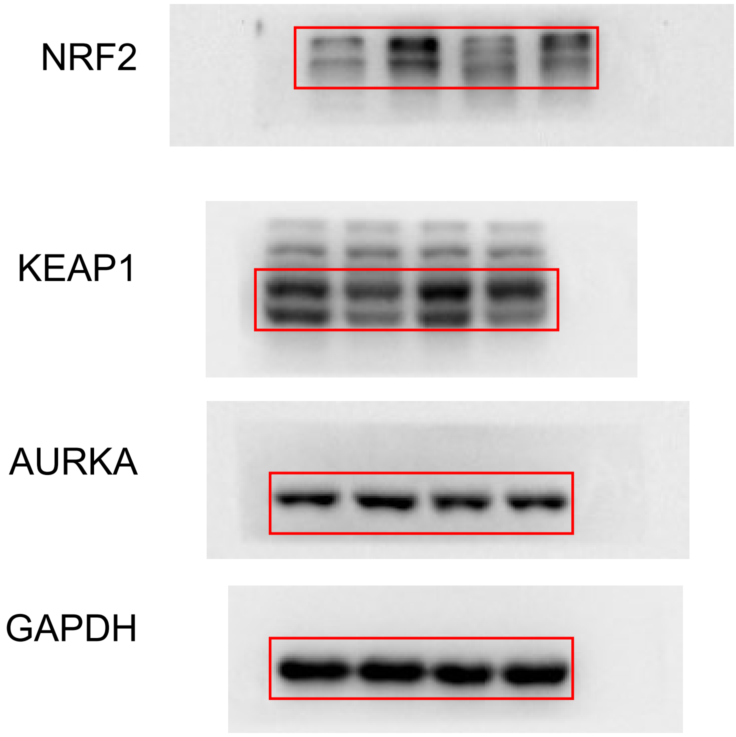

Figure 4A

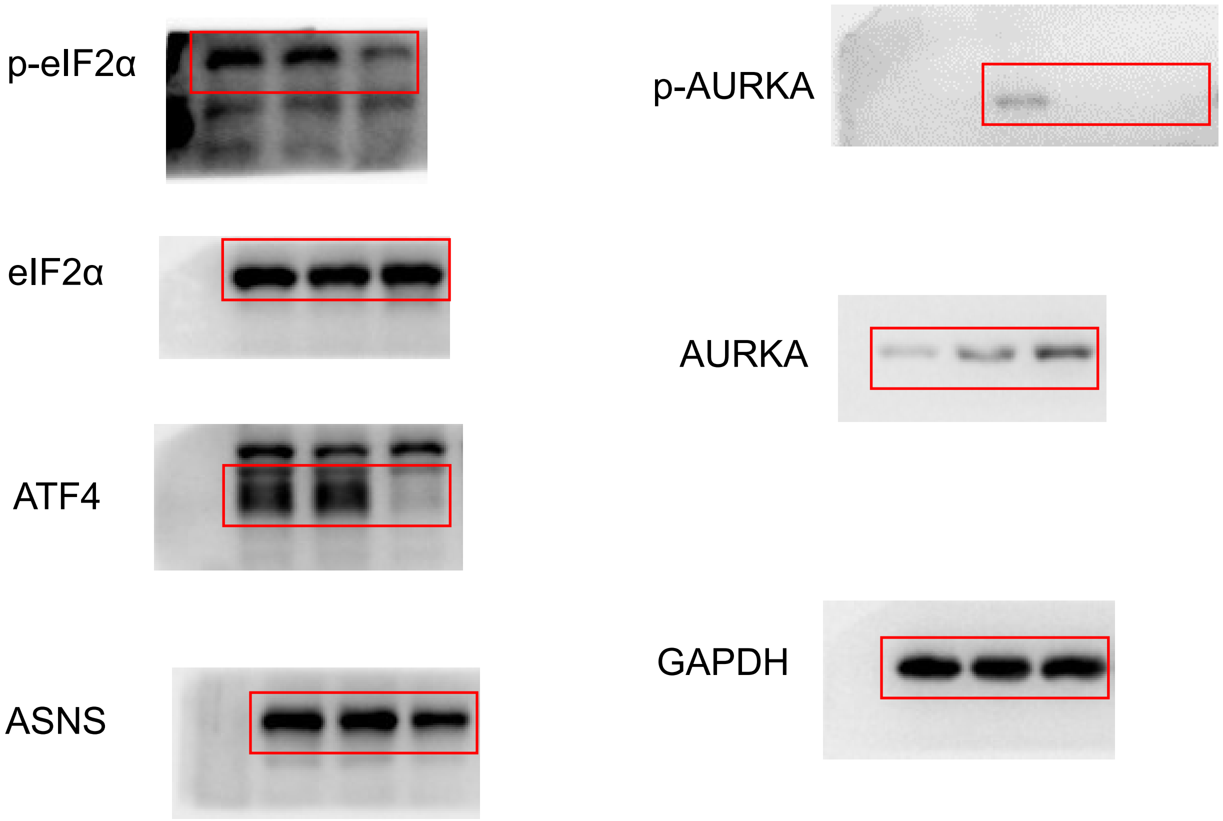

Figure 4B

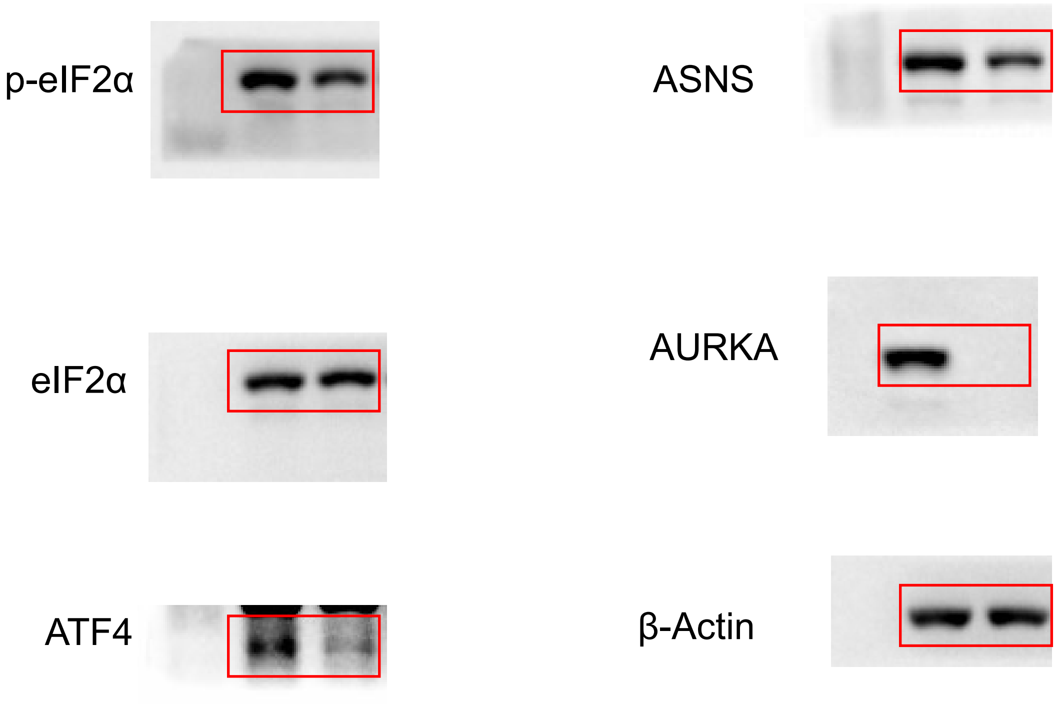

Figure 4D

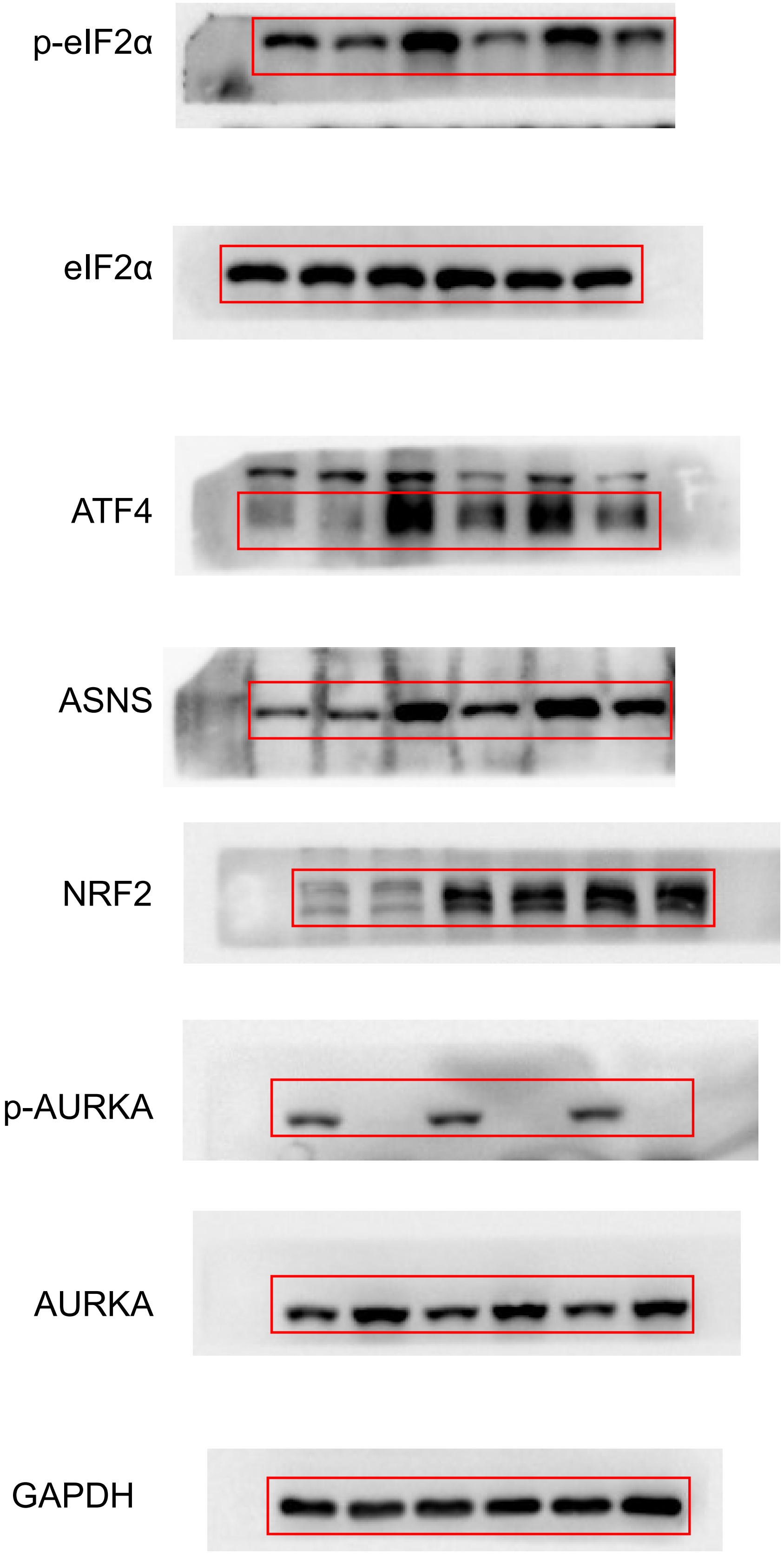

Figure 4G

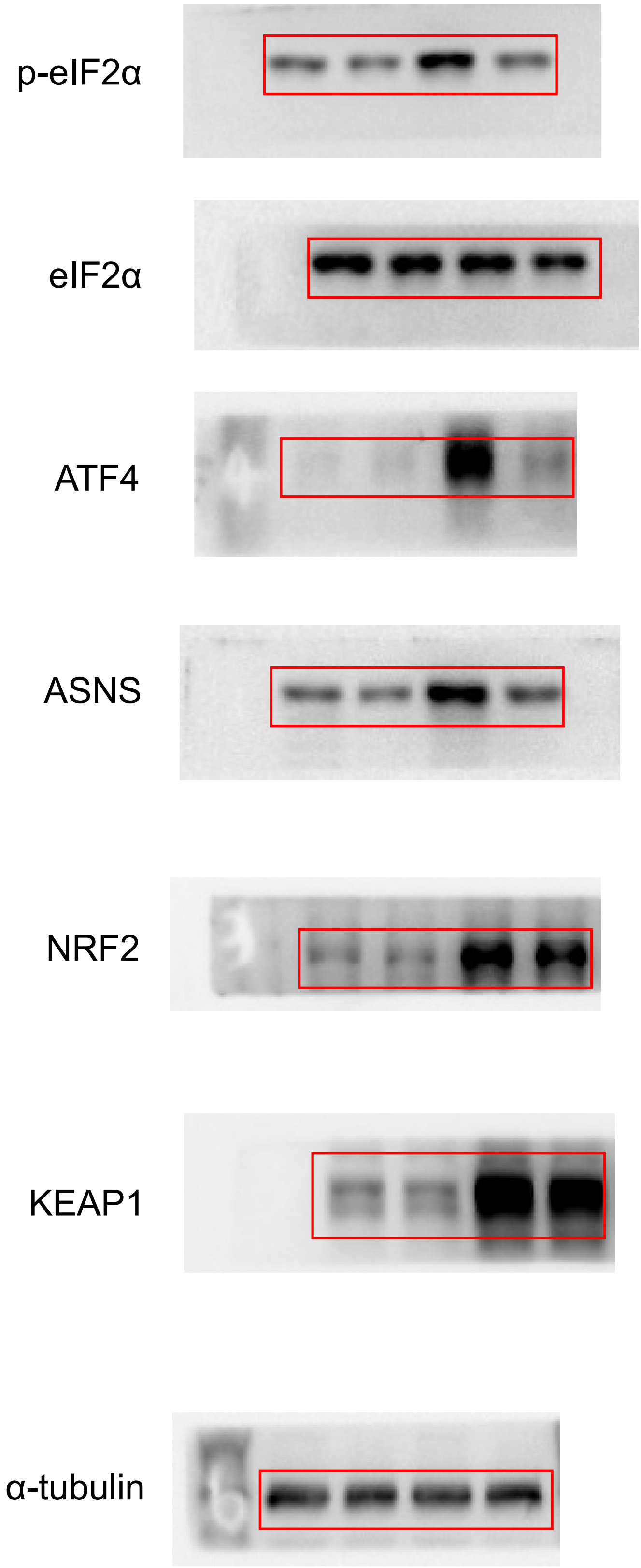

Figure 5A

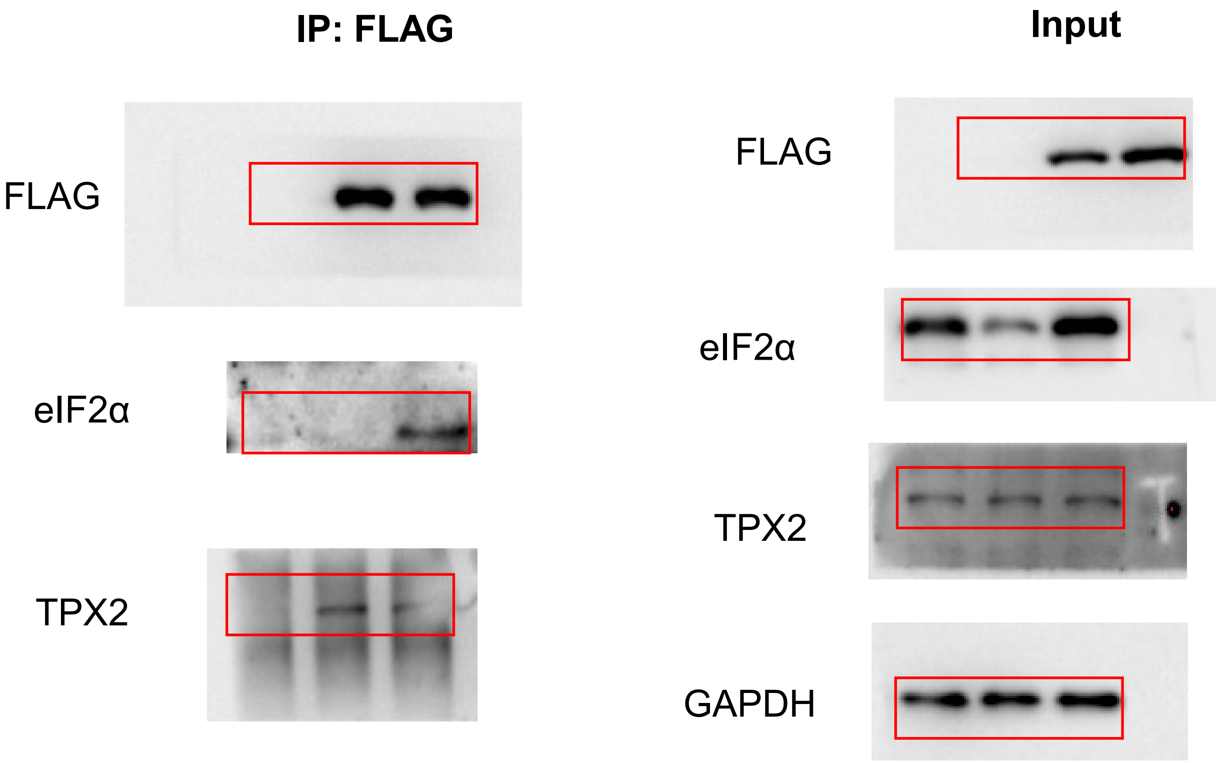

Figure 5B

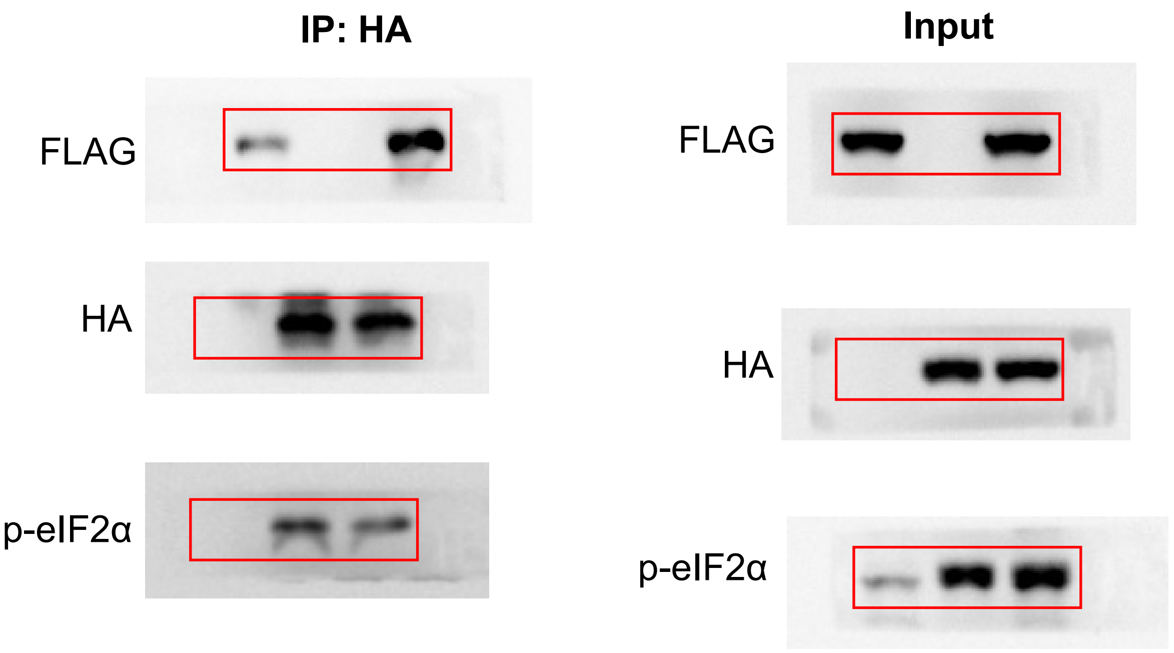

Figure 5C

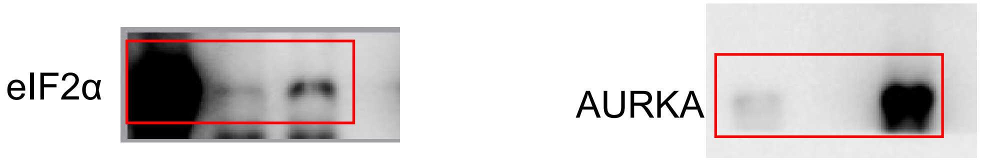

Figure 5D

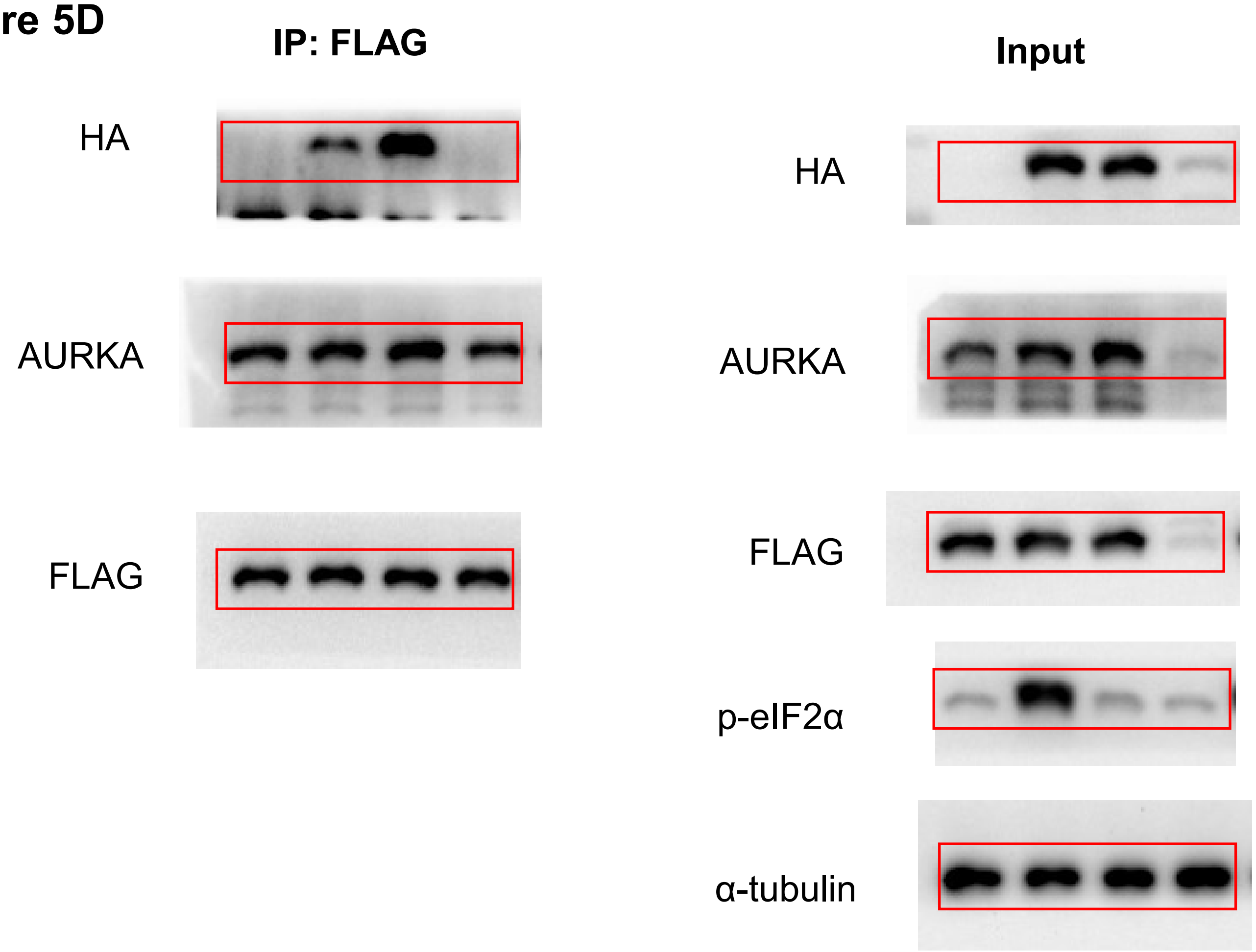

Figure 5E

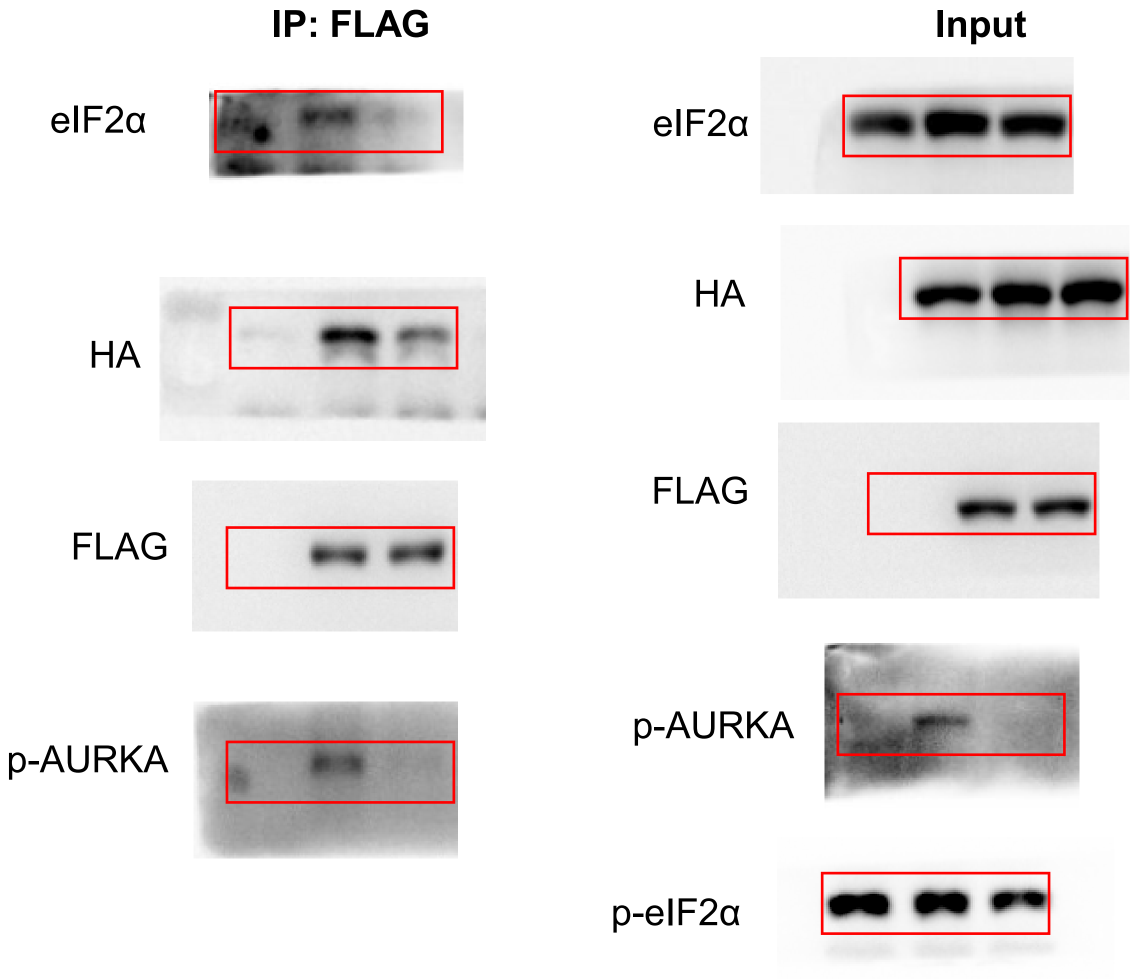

Figure 5F

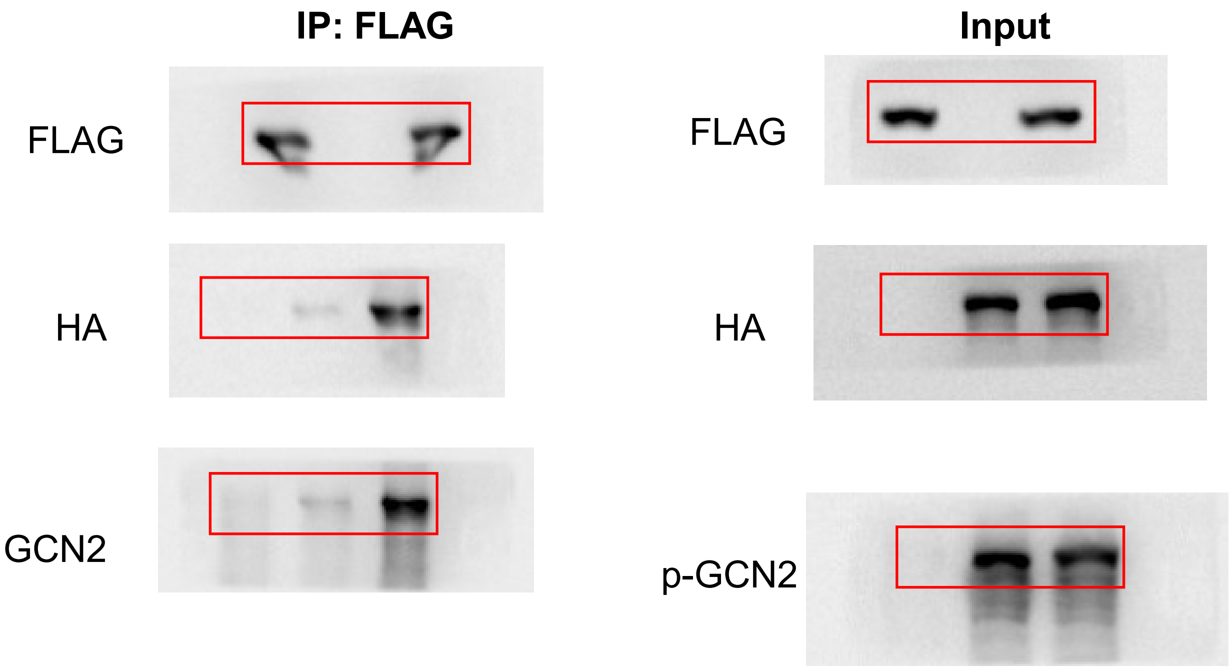

Figure 5G

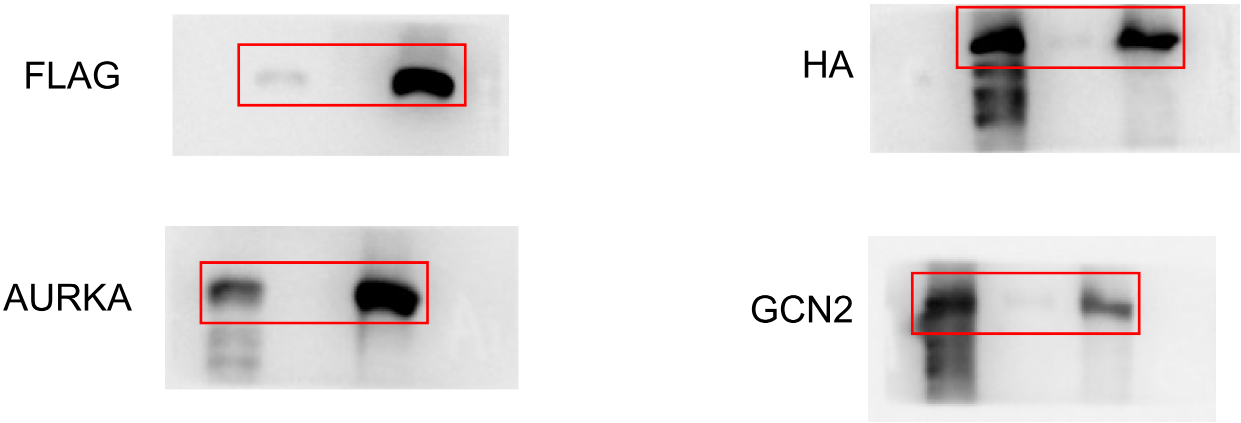

Figure 5H

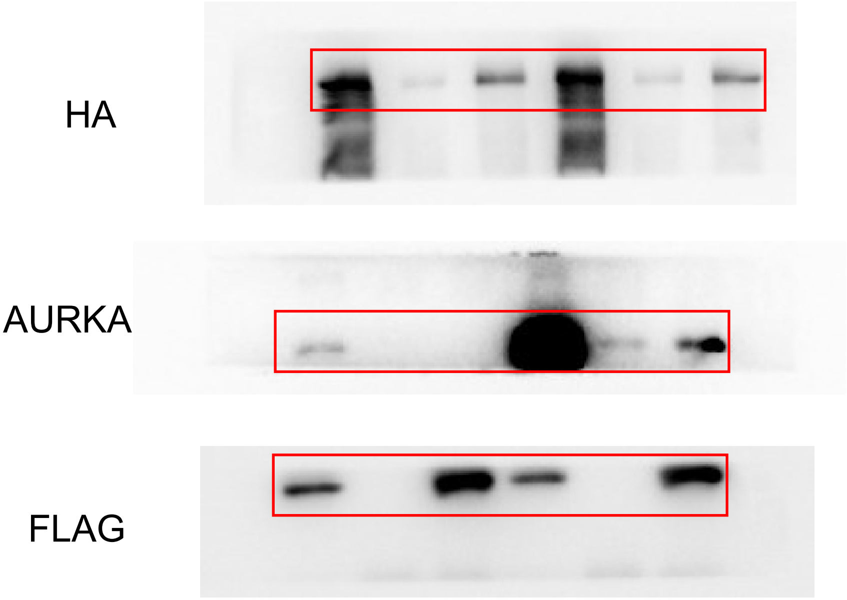

Figure 5I

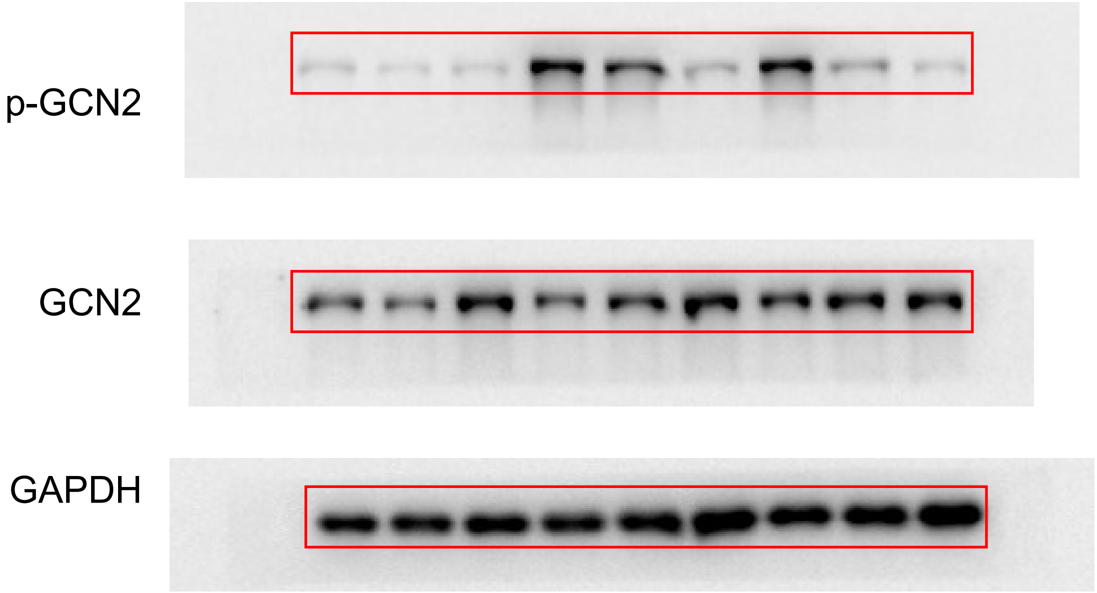

Figure 5J

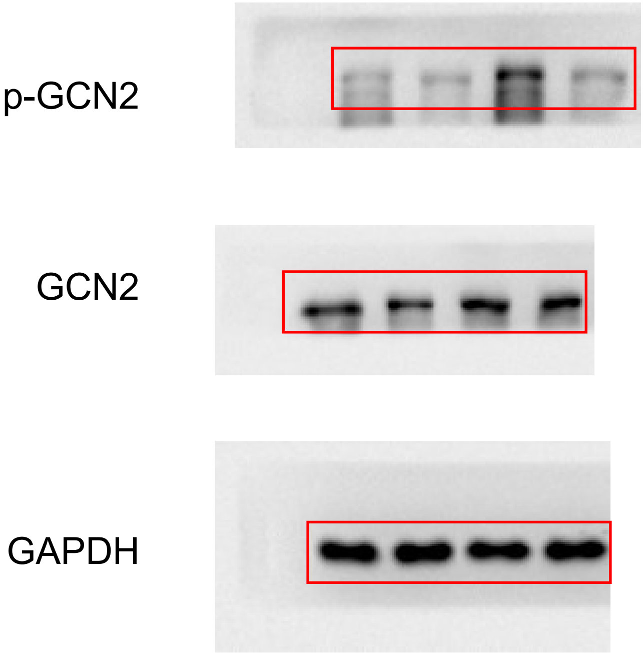

Figure 6A

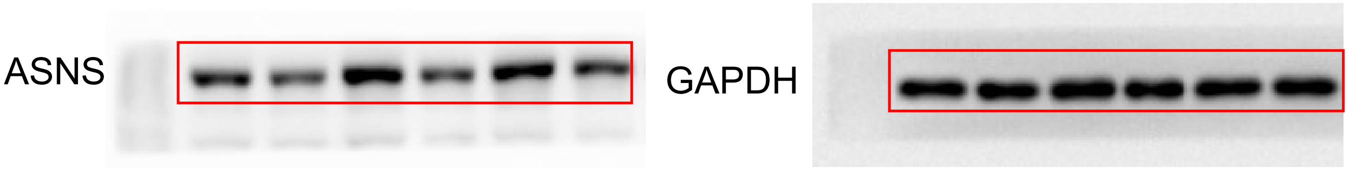

Figure 6D

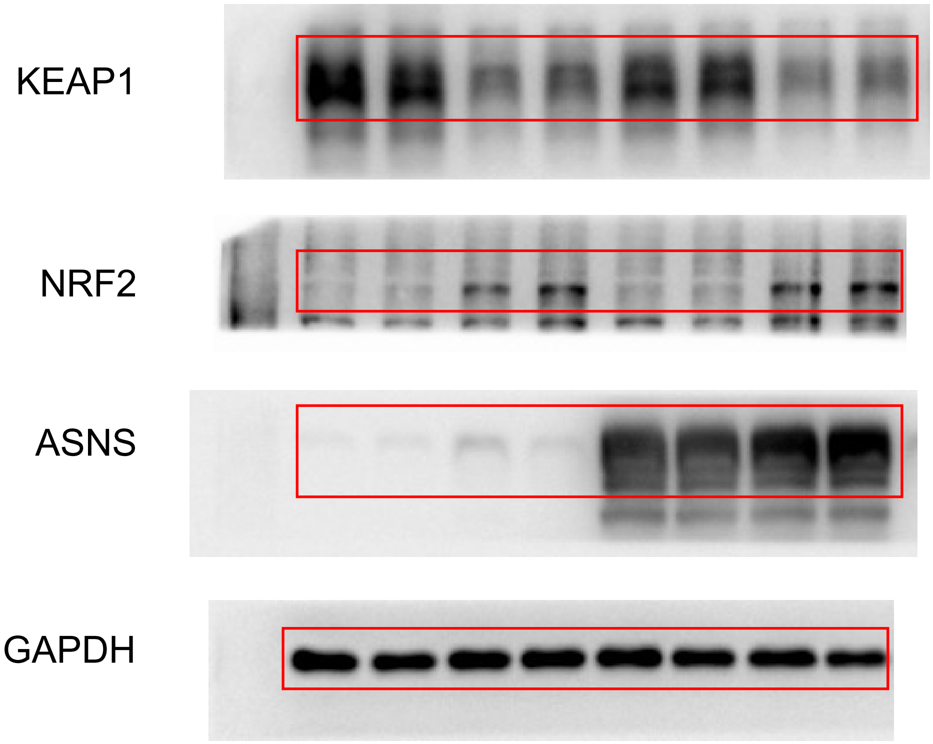

Figure 6E

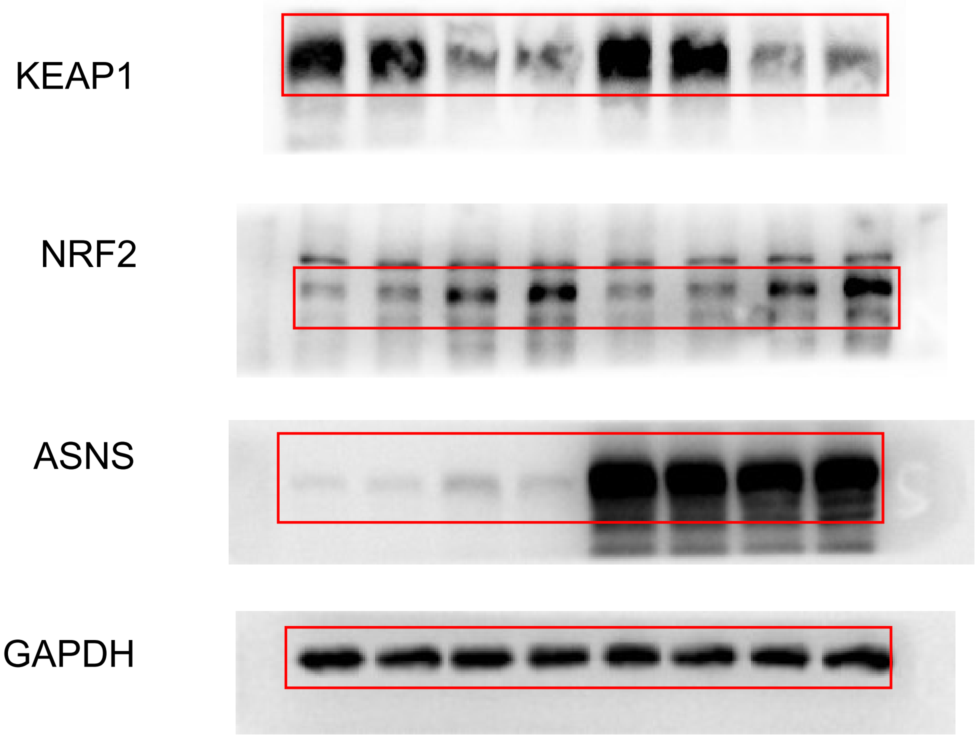

Figure S2D

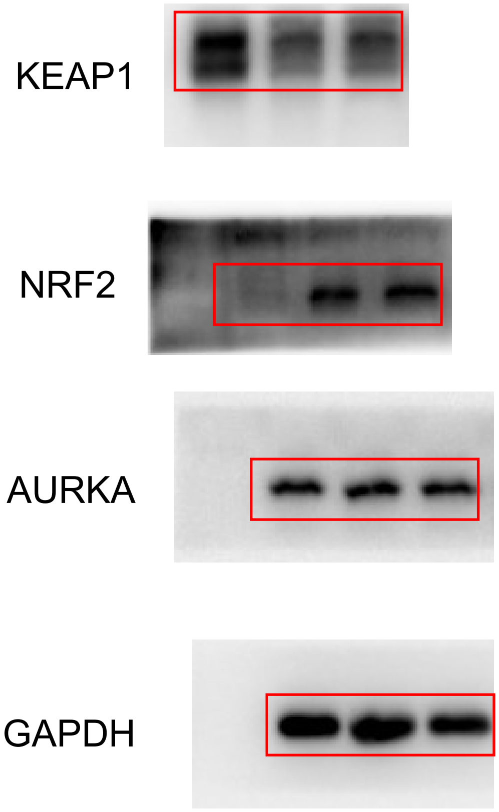

Figure S2H

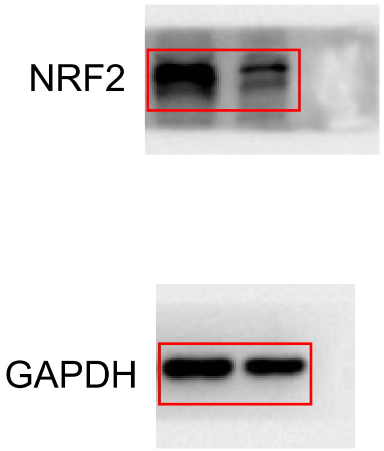

Figure S4A

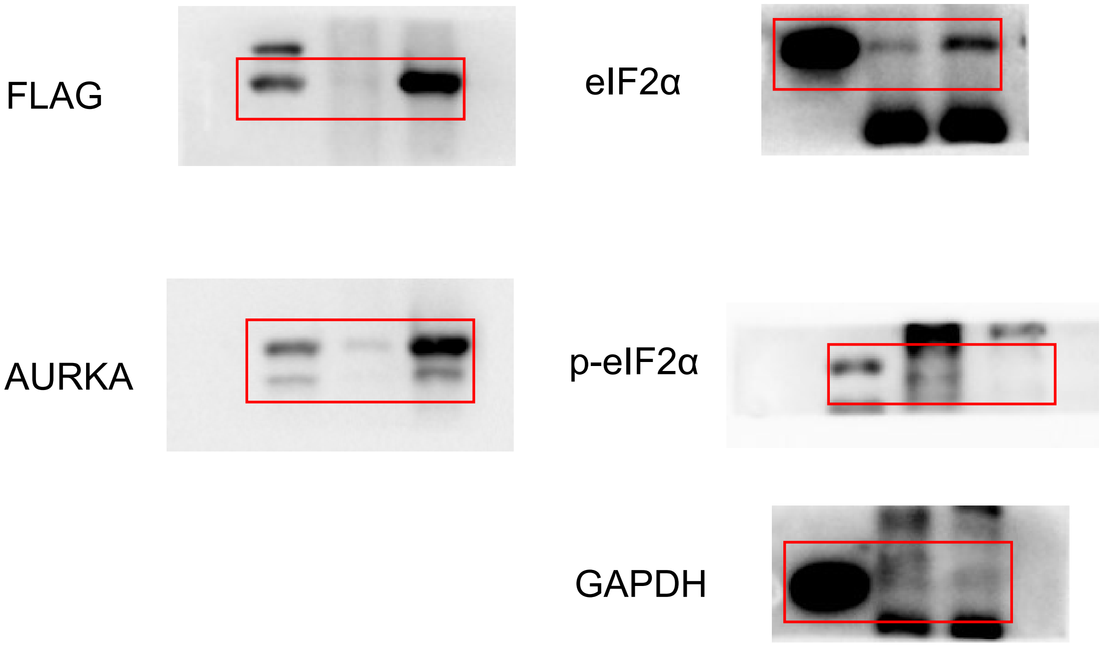

Figure S4B

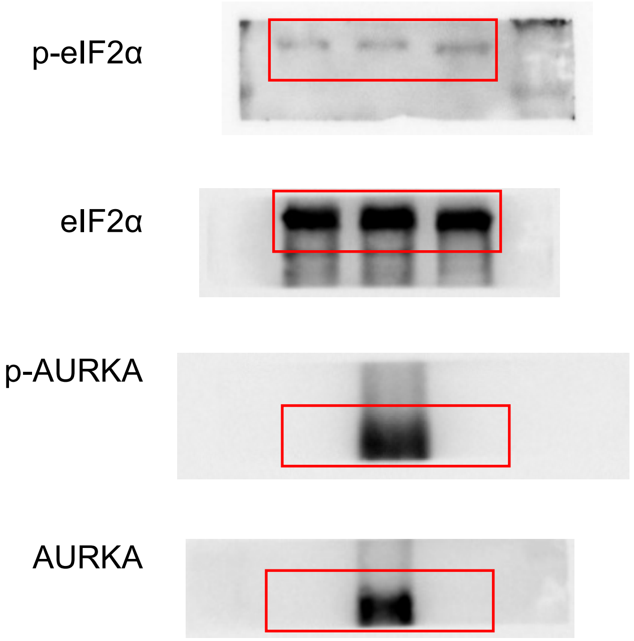

Figure S4C

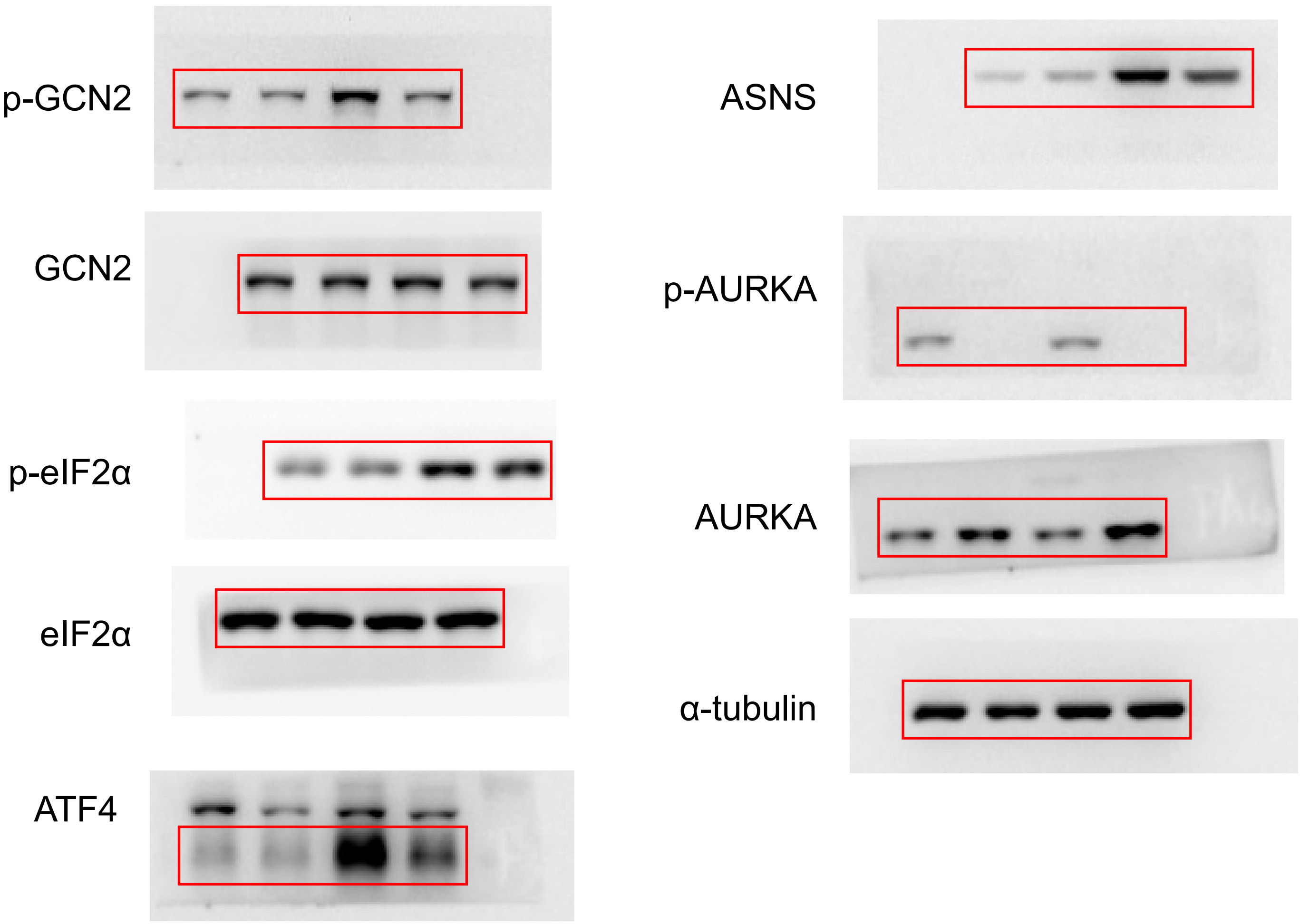

Figure S4D

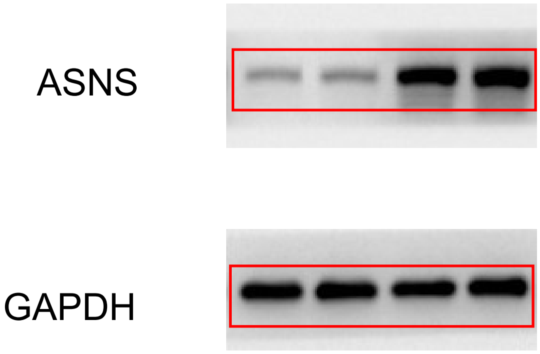

Figure S5A

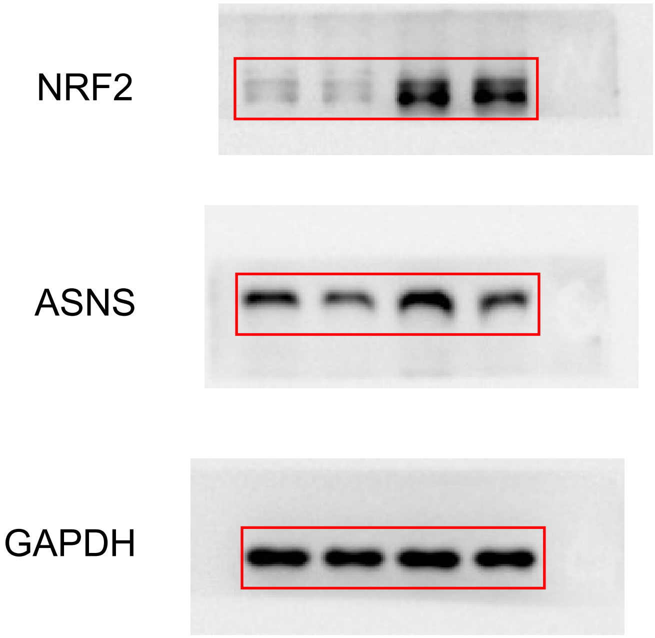

Figure S5F

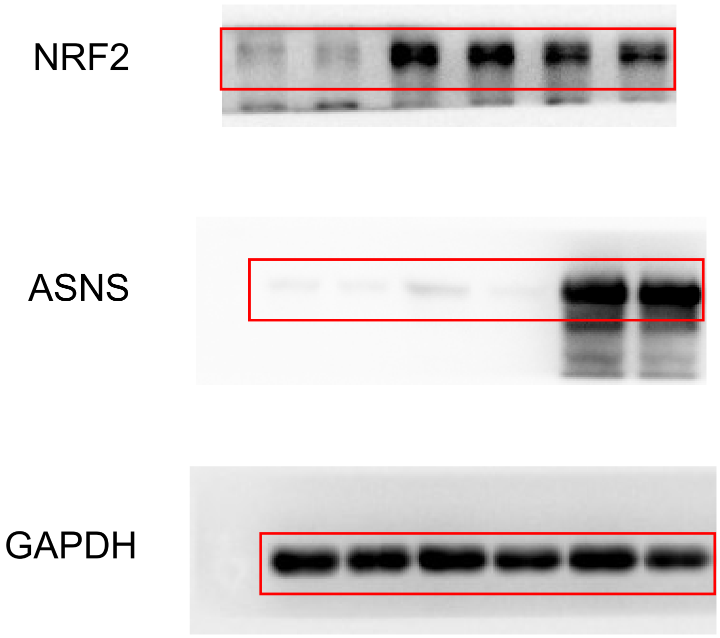

Supplement: Supplementary file 8 — Original Data File [file 41419_2024_6577_MOESM8_ESM.pdf]
